# Overcoming Symmetry Mismatch in Vaccine Nanoassembly through Spontaneous Amidation

**DOI:** 10.1002/anie.202009663

**Published:** 2020-10-27

**Authors:** Rolle Rahikainen, Pramila Rijal, Tiong Kit Tan, Hung‐Jen Wu, Anne‐Marie C. Andersson, Jordan R. Barrett, Thomas A. Bowden, Simon J. Draper, Alain R. Townsend, Mark Howarth

**Affiliations:** ^1^ Department of Biochemistry University of Oxford South Parks Road Oxford OX1 3QU UK; ^2^ MRC Human Immunology Unit MRC Weatherall Institute of Molecular Medicine Radcliffe Department of Medicine University of Oxford Oxford OX3 9DS UK; ^3^ Current address: InProTher Aps Ole Maaløes Vej 3 2200 København Denmark; ^4^ Jenner Institute University of Oxford Oxford OX3 7DQ UK; ^5^ Wellcome Trust Centre for Human Genetics University of Oxford Oxford OX3 7BN UK

**Keywords:** bioconjugation, nanoparticle, nanotechnology, SpyTag, vaccines

## Abstract

Matching of symmetry at interfaces is a fundamental obstacle in molecular assembly. Virus‐like particles (VLPs) are important vaccine platforms against pathogenic threats, including Covid‐19. However, symmetry mismatch can prohibit vaccine nanoassembly. We established an approach for coupling VLPs to diverse antigen symmetries. SpyCatcher003 enabled efficient VLP conjugation and extreme thermal resilience. Many people had pre‐existing antibodies to SpyTag:SpyCatcher but less to the 003 variants. We coupled the computer‐designed VLP not only to monomers (SARS‐CoV‐2) but also to cyclic dimers (Newcastle disease, Lyme disease), trimers (influenza hemagglutinins), and tetramers (influenza neuraminidases). Even an antigen with dihedral symmetry could be displayed. For the global challenge of influenza, SpyTag‐mediated display of trimer and tetramer antigens strongly induced neutralizing antibodies. SpyCatcher003 conjugation enables nanodisplay of diverse symmetries towards generation of potent vaccines.

## Graphical abstract

Nanoassembly can play a key role in generating effective vaccines for emerging (SARS‐CoV‐2) and established (influenza) pandemic threats. SpyTag‐mediated display was used to adapt the nanocage to the challenge of bacterial or viral antigens with diverse cyclic and dihedral symmetries. Establishing display of both trimeric and tetrameric antigens may lead to an influenza vaccine with broader protection.

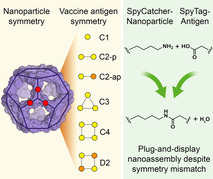

## Introduction

As nanostructures grow and develop, there are often mismatches in size and symmetry at interfaces, including for crystal lattices,^[1]^ metal‐organic frameworks^[2]^ and natural biological assemblies.^[3]^ For example, Adenovirus has trimeric fibers extending from a capsid vertex with C5 (5‐fold cyclic) symmetry, while Salmonella's injectisome has a C24 inner ring connecting to a C15 outer ring.^[3]^ Artificial protein nanostructures have shown great potential for therapy,^[4]^ energy harvesting^[5]^ and vaccines for global health,[^6^, ^7^] so it is important to establish approaches to overcome symmetry mismatch. Vaccines based on purified protein antigens generally have excellent safety profiles, while their facile manufacturing allows rapid response to antigenic variation and emerging pandemic threats.^[8]^ However, such protein antigens show mediocre immunogenicity unless displayed on virus‐like particles (VLPs).^[8]^ VLP‐display facilitates antigen drainage to lymph nodes, complement activation, uptake by antigen‐presenting cells, and B cell receptor crosslinking.^[8]^ However, genetic fusion of antigens to VLP subunits is hindered by different requirements for folding and post‐translational modifications, as well as symmetry mismatches.^[9]^

**Figure 1:**
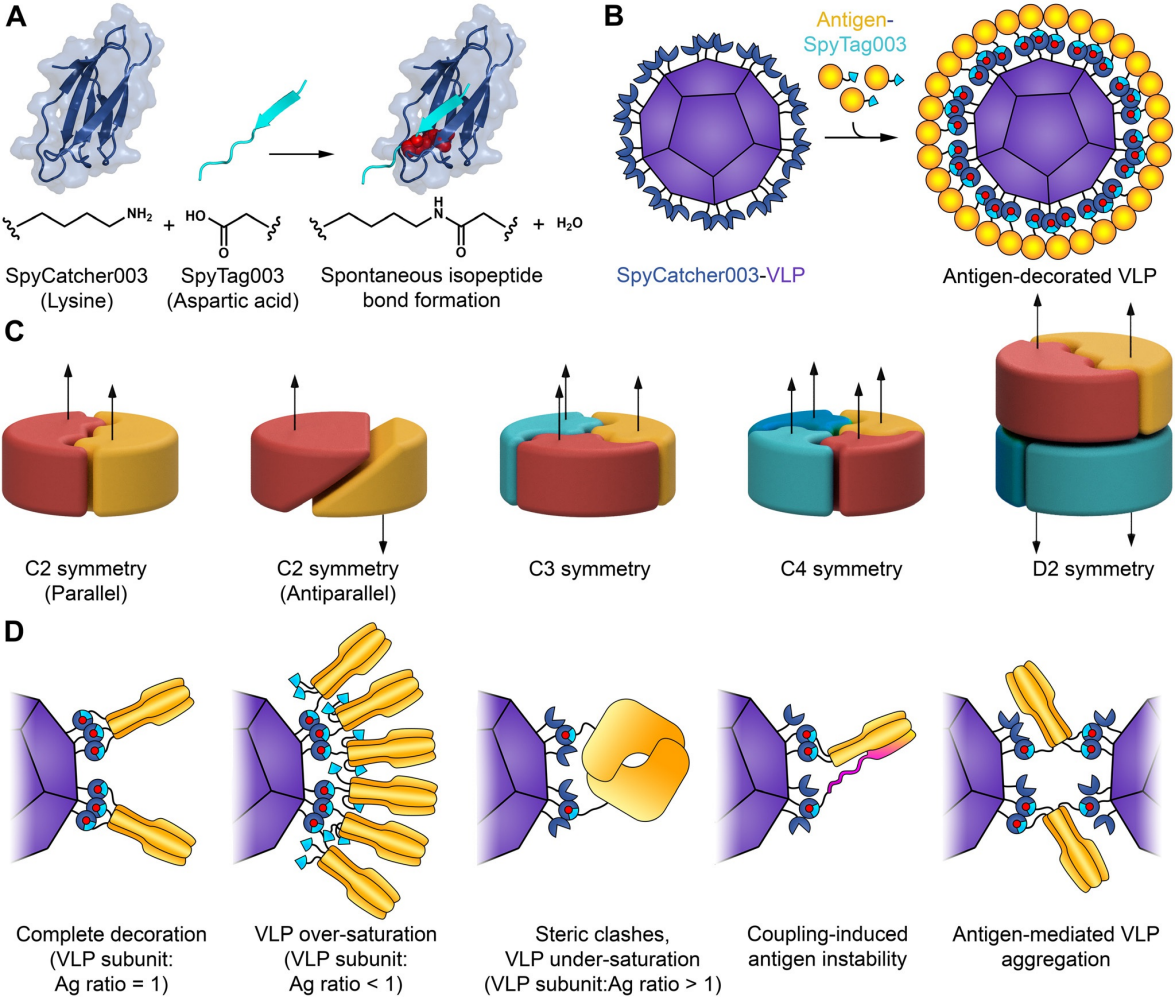
Spy‐nanoassembly for different antigen symmetries. A) Schematic of reactive protein/peptide pair. Lysine on SpyCatcher003 (dark blue) forms a spontaneous amide bond with aspartic acid of SpyTag003 (cyan). Residues forming the isopeptide bond are colored as red spheres, based on PDB ID: 4MLI. B) Plug‐and‐display vaccine assembly. Genetic fusion of a monomeric antigen (yellow) to SpyTag003 (cyan) allows simple efficient reaction (isopeptide bond in red) on nanoparticles (purple) fused to SpyCatcher003 (dark blue). For clarity, only a portion of SpyCatcher003 sites are shown. C) Typical symmetries of multimeric protein antigens (arrow indicates relative subunit orientation). D) Matching of nanoparticle and antigen symmetry. VLP‐display of multimeric antigen (in orange) may proceed to different display densities. Coupling of multimeric antigens could destabilize the antigen (misfolding in magenta) or bridge VLPs, leading to aggregation.

Modular assembly of antigen‐decorated VLPs through SpyTag/SpyCatcher technology represents an alternative avenue.^[9]^ SpyCatcher is a 12 kDa protein that we previously engineered to form a spontaneous amide bond to its peptide partner SpyTag (Figure 1 A).^[10]^ While the display of monomeric antigens using modular ligation (Figure 1 B) is established,[^11^, ^12^, ^13^] there has been no systematic study of the display of antigens possessing different cyclic or dihedral symmetries (Figure 1 C). However, a large fraction of clinically important antigens are multimeric, including surface proteins from influenza, Ebola virus and coronaviruses.[^6^, ^7^, ^8^] Potential challenges for VLP‐display of multimeric antigens include unpredictable antigen density on the VLP, antigen instability, or VLP crosslinking leading to aggregation (Figure 1 D).

Here, we establish a VLP showing efficient production, quantitative amide bond formation, and extreme resilience. We characterize VLP reactivity with multimeric antigens of diverse symmetry, spanning a range of important human and veterinary pathogens. The level of pre‐existing human immunity against different SpyTag/SpyCatcher generations is investigated using serum from healthy donors. Finally, we establish the new platform to overcome the obstacle of display of both trimeric and tetrameric influenza antigens, enabling induction of potent neutralizing antibody responses.

## Results

**Figure 2:**
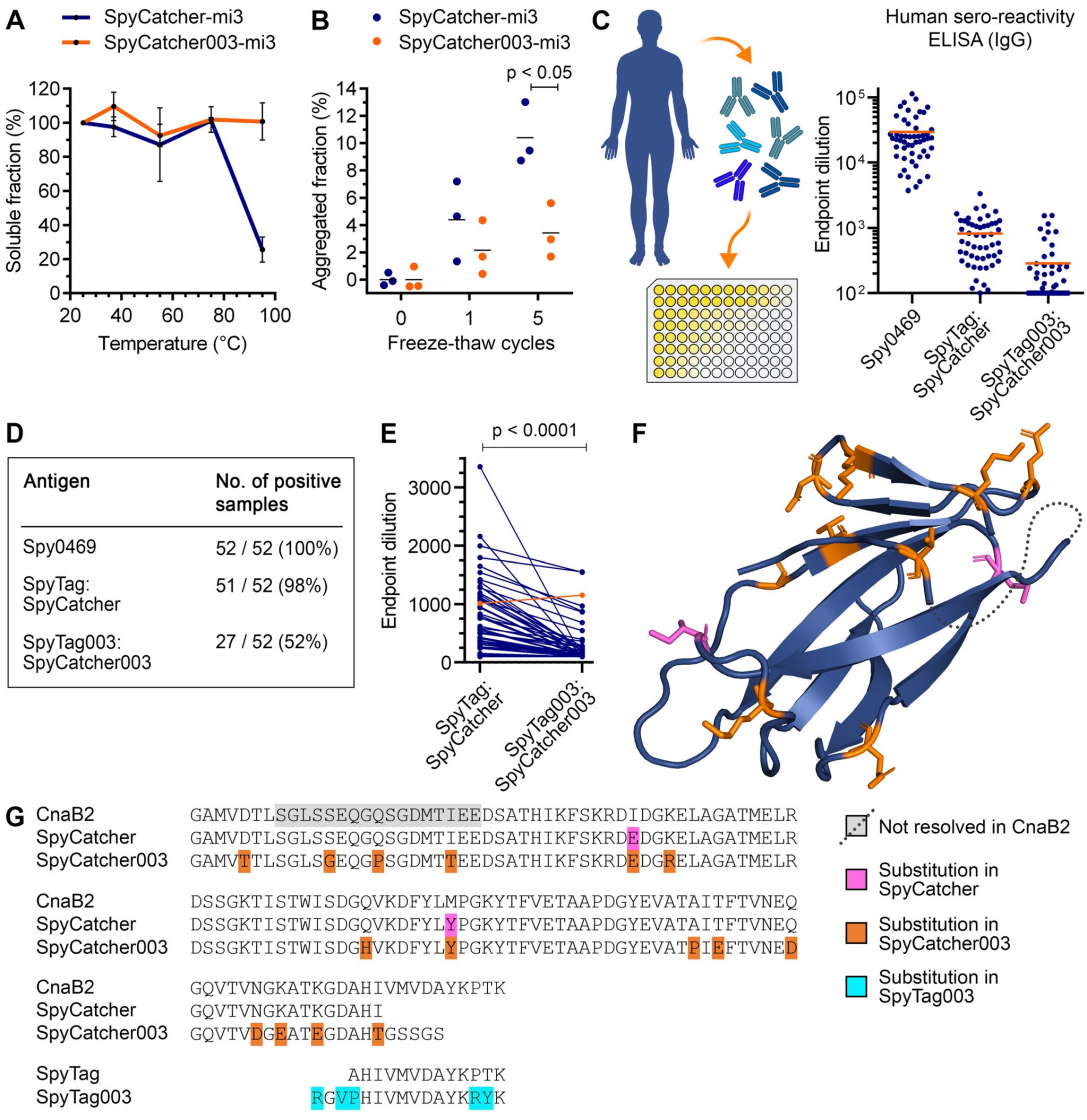
SpyCatcher003 enhanced VLP stability and decreased pre‐existing sero‐reactivity. A) SpyCatcher‐mi3 and SpyCatcher003‐mi3 were heated for 60 min at the indicated temperature, cooled to 4 °C, aggregates were removed by centrifugation, and the fraction of soluble protein determined. Mean±s.d., *n*=3. B) SpyCatcher‐mi3 or SpyCatcher003‐mi3 were subjected to the indicated number of freeze‐thaw cycles and the insoluble fraction was removed by centrifugation. VLP in the supernatant was analyzed by SDS‐PAGE. Mean is marked by a horizontal line; two‐tailed Student's t‐test, *n*=3. C) Pre‐existing sero‐reactivity to *S. pyogenes* antigens. Antibody titer of serum from 52 people against the positive control Spy0469, SpyTag:SpyCatcher, or SpyTag003:SpyCatcher003. Each person is shown as a dot, with the mean in orange. D) Summary of immune responses in (C). E) Pairwise comparison of titer. The person with increased titer against SpyTag003:SpyCatcher003 is indicated in orange (Wilcoxon matched‐pairs signed rank test, *n*=52). F–G) Structural model (PDB ID: 2X5P) of the CnaB2 domain and sequence alignment with SpyTag/SpyCatcher pairs. Substitutions from CnaB2 are magenta (SpyCatcher) or orange (SpyCatcher003). Substitutions in SpyTag003 are cyan. The dotted line indicates a region not resolved in CnaB2.

Initially we established an optimal nanoparticle scaffold for vaccine assembly. The Baker laboratory designed a dimeric interface into the trimeric aldolase from the thermophilic bacterium *Thermotoga maritima*, leading to porous 60‐mer dodecahedra.^[14]^ We previously increased the resilience and modular reactivity of these particles to generate SpyCatcher‐mi3.^[15]^ In parallel, we recently developed SpyTag003/SpyCatcher003, with reaction rate near the diffusion limit.^[16]^ Therefore, we solubly expressed SpyCatcher003‐mi3 VLPs in the *Escherichia coli* cytoplasm to high yield and investigated their biophysical and biochemical properties. The original SpyCatcher‐mi3 was purified using C‐tag affinity chromatography. Since affinity‐based purification increases cost and slows scaling,^[17]^ we established affinity resin‐independent purification. Combining ammonium sulfate precipitation and size exclusion chromatography (SEC) allowed efficient purification of SpyCatcher003‐mi3 particles to high purity (Figure S1A–C). Given the great problem of the failure of the cold‐chain for global vaccine usage,^[18]^ we used thermal and freeze‐thaw stability analyses as surrogate assays to assess the resilience of the new VLP platform. SpyCatcher003‐mi3 maintained solubility even following 1 h incubation at 95 °C, exhibiting superior thermal resilience to the original SpyCatcher‐mi3 platform (Figure 2 A). Similarly, SpyCatcher003‐mi3 showed enhanced stability to freeze‐thaw compared to SpyCatcher‐mi3 (Figure 2 B).

SpyTag‐mediated amidation has been tested in a range of pre‐clinical vaccine models, particularly for malaria,^[9]^ but it has not been explored to what extent people have pre‐existing antibodies against SpyTag/SpyCatcher. SpyTag/SpyCatcher was engineered from the CnaB2 domain of the FbaB adhesion protein from the common human pathogen *Streptococcus pyogenes*.^[10]^ *S. pyogenes* causes infection in 700 million people worldwide per year.^[19]^ FbaB is predominantly expressed by the M3 serotype of *S. pyogenes*.^[20]^ To better understand the implications of common exposure to *S. pyogenes* for clinical development of SpyTag/SpyCatcher‐based vaccines, we used enzyme‐linked immunosorbent assay (ELISA) to analyze the presence of pre‐existing antibodies against SpyTag:SpyCatcher and SpyTag003:SpyCatcher003 in serum samples from 52 healthy UK adults. Each Catcher was pre‐reacted with the respective Tag and purification tags were removed (Figure S2A), to mimic the conjugates present on antigen‐decorated VLPs. All 52 people tested positive for antibodies against the immunodominant *S. pyogenes* surface protein Spy0469,^[19]^ confirming the expected common exposure of people to *S. pyogenes* (Figure 2 C,D, Figure S2B). Antibodies to SpyTag:SpyCatcher were detected in 98 % of samples, but only 52 % gave a positive response to SpyTag003:SpyCatcher003 (5.4‐fold decrease in median titer, Figure 2 C,D, Figure S2C,D). In pairwise comparison only 1/52 had higher titer against SpyTag003:SpyCatcher003 (Figure 2 E). Therefore, the 18 amino acid substitutions from SpyTag:SpyCatcher to SpyTag003:SpyCatcher003^[16]^ generally decreased detection by existing human antibodies (Figure 2 F,G). We confirmed this difference using sandwich ELISA with anti‐His_6_ antibody (Figure S2E,F). We investigated if high anti‐Spy0469 titer predicts high anti‐SpyCatcher response. Spy0469 versus SpyTag:SpyCatcher had a Pearson correlation coefficient of 0.33 (*p*=0.02, *n*=51), indicating a weak positive relationship (Figure S2G). However, we did not find a significant correlation between the Spy0469 and SpyTag003:SpyCatcher003 titres (Pearson correlation coefficient=0.28, *p*=0.15, *n*=27) (Figure S2 H). As expected, SpyTag:SpyCatcher and SpyTag003:SpyCatcher003 titers had a statistically significant positive correlation (Pearson correlation coefficient=0.67, *p*=0.0001, *n*=27) (Figure S2I).

We have also established SnoopTag/SnoopCatcher as an orthogonal reactive pair for spontaneous transamidation and vaccine nanoassembly^[21]^ (Figure S3A). SnoopTag/SnoopCatcher was engineered from the RrgA adhesin of *Streptococcus pneumoniae*; up to 65 % of children and 10 % of adults are asymptomatic carriers.^[22]^ All human sera tested positive against *S. pneumoniae* immunodominant surface protein PsaA^[23]^ (Figure S3B,C,E,F). Only 25 % were positive against SnoopTag/SnoopCatcher (Figure S3D‐F). We did not find correlation between PsaA and SnoopTag:SnoopCatcher titers (Pearson correlation coefficient=0.15, *p*=0.63, *n*=13) (Figure S3G).

**Figure 3:**
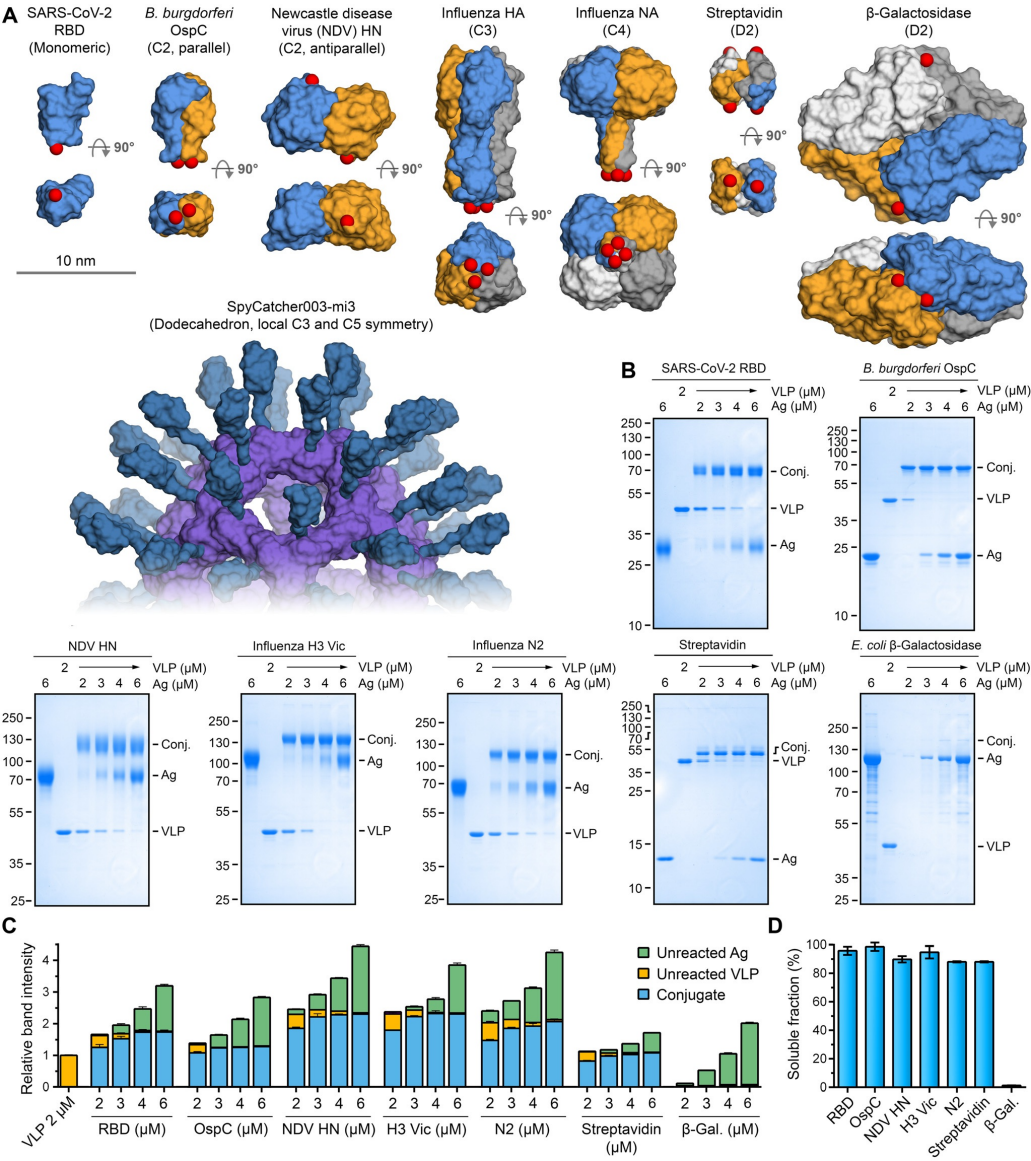
SpyCatcher003‐mi3 particles allowed efficient display of antigens with different sizes and symmetries. A) Structures of the antigens with varying symmetry. Each antigen is shown in two orientations with space‐filling model, with each chain in a different color. A model of SpyCatcher003‐mi3 is presented at the same scale. PDB IDs: RBD: 6W41, OspC: 1G5Z, NDV HN: 3T1E, HA: 4UO0, NA: 2HTY (with tetramerization domain: 1USE), streptavidin: 1SWB, β‐galactosidase: 6DRV. Red spheres indicate where the reactive peptide is fused. B) Coupling of model antigens at different molar VLP:antigen ratios. Samples were reacted for 16 h at 4 °C, insoluble aggregates removed by centrifugation, and supernatant loaded on reducing SDS‐PAGE before Coomassie staining. C) Quantification of VLP:antigen reactions. The intensity of each band from gel densitometry was divided by the intensity of the band of VLP alone at 2 μM. Bars represent mean+s.d., *n*=3. D) Antigens with cyclic symmetry maintained good VLP solubility. Soluble fraction of antigens conjugated at 1:1 molar ratio was determined by densitometry (mean±s.d., *n*=3). Some error bars are too small to be visible.

To investigate VLP‐display of antigens with diverse sizes and multimeric structures, we expressed a panel of SpyTag‐ and SpyTag003‐fused proteins (Figure 3 A). As a monomer, we expressed the receptor binding domain (RBD) from SARS‐CoV‐2 spike (Figure 3 A), a vaccine candidate for Covid‐19,^[24]^ bearing an N‐terminal SpyTag.

Our first dimer was Outer surface protein C (OspC), an important vaccine candidate from *Borrelia burgdorferi*, the bacterium causing Lyme disease. OspC is expressed both during transmission from the tick and upon entry into the mammalian host.^[25]^ We expressed an N‐terminally truncated fragment of OspC solubly in *E. coli* with SpyTag003 at its N‐terminus. The OspC Δ19 C129S fragment, a parallel dimer (C2 symmetry, Figure 3 A), is highly immunogenic and induces protective immunity against *B. burgdorferi* infection.^[25]^

Newcastle Disease Virus (NDV) is a paramyxovirus and a particular danger for poultry farming. NDV's hemagglutinin‐neuraminidase (HN) is crucial in host‐cell entry and egress and, like many paramyxovirus antigens, exists as a loosely‐associated tetramer of two approximately antiparallel dimers.[^26^, ^27^] We expressed an N‐terminally truncated fragment of HN (termed NDV HN), aiming to generate a C2 dimer (Figure 3 A) bearing SpyTag003 at its C‐terminus. The two C‐termini of the HN dimer in PDB ID: 3T1E are >7 nm apart.^[26]^

Influenza virus is a persistent challenge for both human and veterinary health.^[28]^ Annual human epidemics of influenza cause ≈3–5 million cases of severe illness, with 290 000–650 000 deaths.^[28]^ Current influenza vaccines may fail to induce potent protection, especially in people over 65.^[29]^ Furthermore, vaccines produced in eggs are slow to scale up and antigen egg‐adaptation can lower effectiveness.^[30]^ Current vaccines mostly induce antibodies to hemagglutinin (HA) but not neuraminidase (NA), even though antibodies to both contribute to protection.^[31]^ As a trimer with C3 symmetry, HA is well suited for genetic fusion to the 3‐fold axis of various protein nanoparticles.[^6^, ^7^] However, NA is a tetramer with C4 symmetry, complicating genetic fusion to self‐assembling protein VLPs. Therefore, we explored whether each of these symmetries could be decorated on our VLP platform. Since influenza shows substantial diversity,^[28]^ we tested our approach on antigens from distinct strains. For HA, we tested H3 from A/Aichi/2/1968 (termed H3 Aichi) and A/Victoria/361/2011 (termed H3 Vic) influenza strains. For NA, we tested N2 from A/Victoria/361/2011 (termed N2) and N1 from A/England/195/2009 (termed N1). As tetramers with dihedral symmetry (D2), we coupled two proteins of divergent size; *Streptomyces avidinii* streptavidin (60 kDa) and the very large *E. coli* β‐galactosidase (483 kDa) (Figure 3 A).

Antigens were conjugated with SpyCatcher003‐mi3 at different molar ratios for 16 h at 4 °C and any insoluble aggregates removed by centrifugation. The amount of covalent VLP‐Antigen conjugate formed at each coupling ratio was quantified using SDS‐PAGE and gel densitometry. Apart from β‐galactosidase, all antigens were efficiently displayed on SpyCatcher003‐mi3 (Figure 3 B,C, Figure S4A,B). When incubated at equimolar concentration of Tag and Catcher, most reactions produced minimal residual non‐conjugated antigen (Figure 3 B,C). This lack of free antigen subunits indicates that at a low antigen concentration, antigen coupling predominantly takes place though all SpyTags on each antigen (Figure 1 D). With all tested antigens, near‐quantitative coupling of all VLP SpyCatchers was achievable (Figure 3 B), suggesting that steric hindrance did not block SpyCatcher003 reactivity even when large multimeric antigens were displayed. However, our panel of antigens suggests that both the size of antigen and orientation of its subunits can have an effect on coupling efficiency. OspC efficiently reacted through both subunits and saturated SpyCatcher003 at low VLP:antigen ratios (Figure 3 B,C). Similarly, the two variants of influenza HA had minimal free subunits left at 1:1 and 1:1.5 molar ratios, indicating that the HA variants were mainly conjugated through all three SpyTags (Figure 3 B,C, Figure S4A,B,C). In contrast, our NDV HN construct, an antiparallel dimer, required 3‐fold antigen excess for VLP saturation, with more non‐covalently coupled subunits present at the saturating level (i.e. coupled through only one tag of each dimer) (Figure 3 B,C). For antigens with dihedral symmetry, streptavidin retained good VLP solubility but β‐galactosidase led to almost complete loss of soluble VLP at all ratios (Figure 3 B‐D). This difference suggests that antigen size is critical when Tags are located on different faces of the antigen. We also established efficient co‐display of HA and NA on VLPs; varying the ratio of antigens during conjugation allowed simple adjustment of display density (Figure S4D). This approach provides a simple way to create vaccine candidates mimicking the density of HA and NA on natural influenza virions.

**Figure 4:**
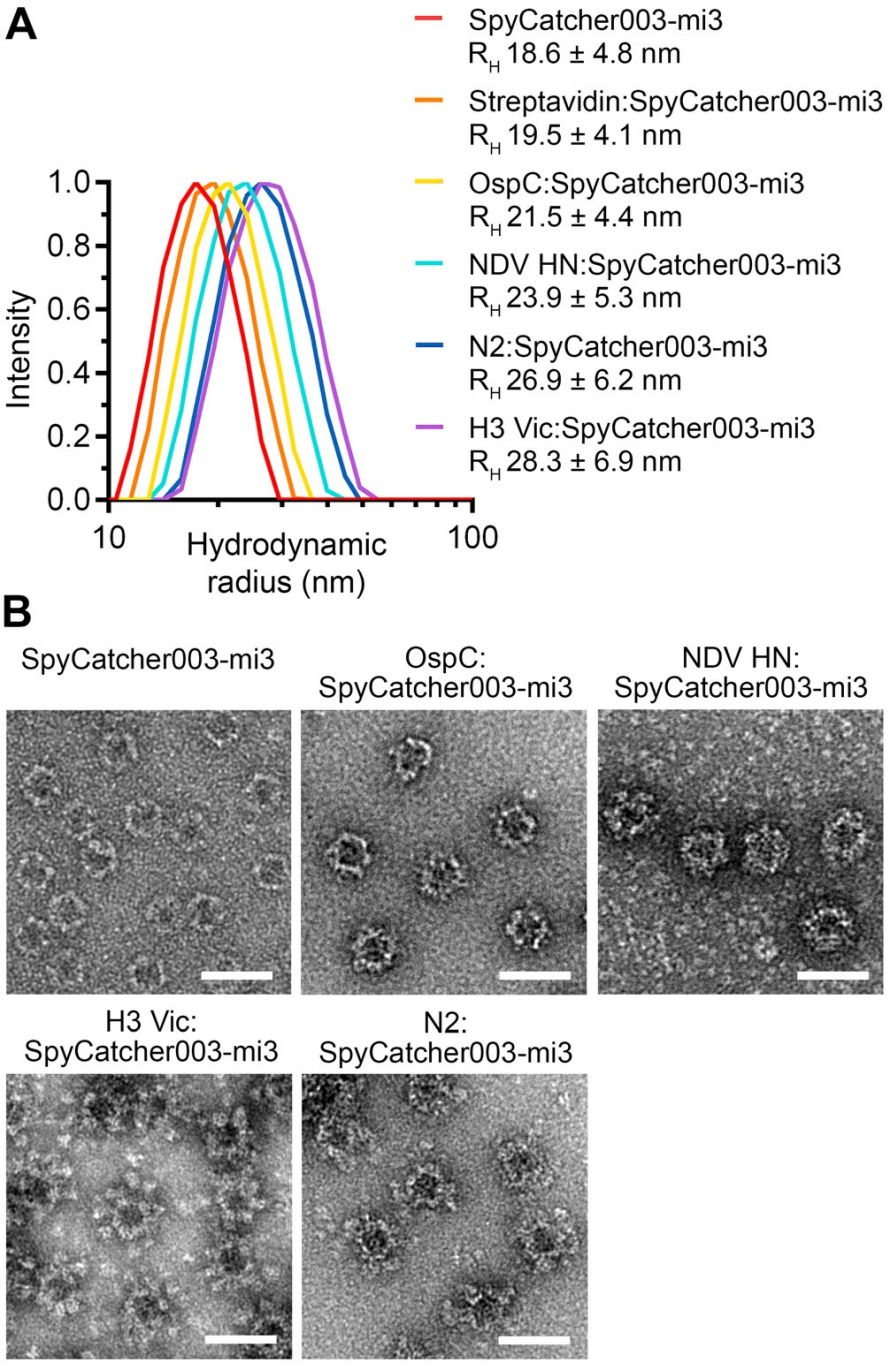
Particle size‐distribution after VLP decoration of antigens of various symmetry. A) Hydrodynamic radius for SpyCatcher003‐mi3 particles, with or without conjugation to antigens (mean±s.d.; for H3 Vic *n*=2 independent experiments; for all other samples *n*=3). B) TEM of negative‐stained antigen‐decorated SpyCatcher003‐mi3 VLPs. Scale‐bar: 50 nm.

Analysis of solubility does not necessarily reveal low levels of VLP crosslinking, structural changes in the VLP, or the presence of soluble aggregates. Therefore, we studied size distribution and monodispersity by dynamic light scattering (DLS). The hydrodynamic radius of non‐conjugated SpyCatcher003‐mi3 was 18.6 nm. The hydrodynamic radius of antigen‐conjugated particles ranged from 19.5 nm (Streptavidin) to 28.3 nm (H3 Vic), consistent with antigen size (Figure 4 A, Figure S5A–G). Both undecorated and antigen‐decorated VLPs had narrow size distributions, indicating that antigen‐decorated VLPs were monodisperse and VLP crosslinking was minimal. As expected, antigen‐decorated particles exhibited distinct shapes and sizes depending on the displayed antigen, by transmission electron microscopy (TEM) after negative‐staining (Figure 4 B). H3 Vic trimers decorated on SpyCatcher003‐mi3 pointed outwards in a radial arrangement, mimicking the orientation of HA on natural influenza virions. To investigate antigen stability after VLP decoration, we studied each dimeric antigen using Thermofluor. OspC assembly on SpyCatcher003‐mi3 resulted in a small increase of 0.9 °C in antigen *T*_m_ (Figure S6A). NDV HN decoration on SpyCatcher003‐mi3 increased antigen *T*_m_ by 3.2 °C (Figure S6B). Therefore, Spy‐VLP display maintains good antigen integrity and can even stabilize the structure of some multimeric antigens.

**Figure 5:**
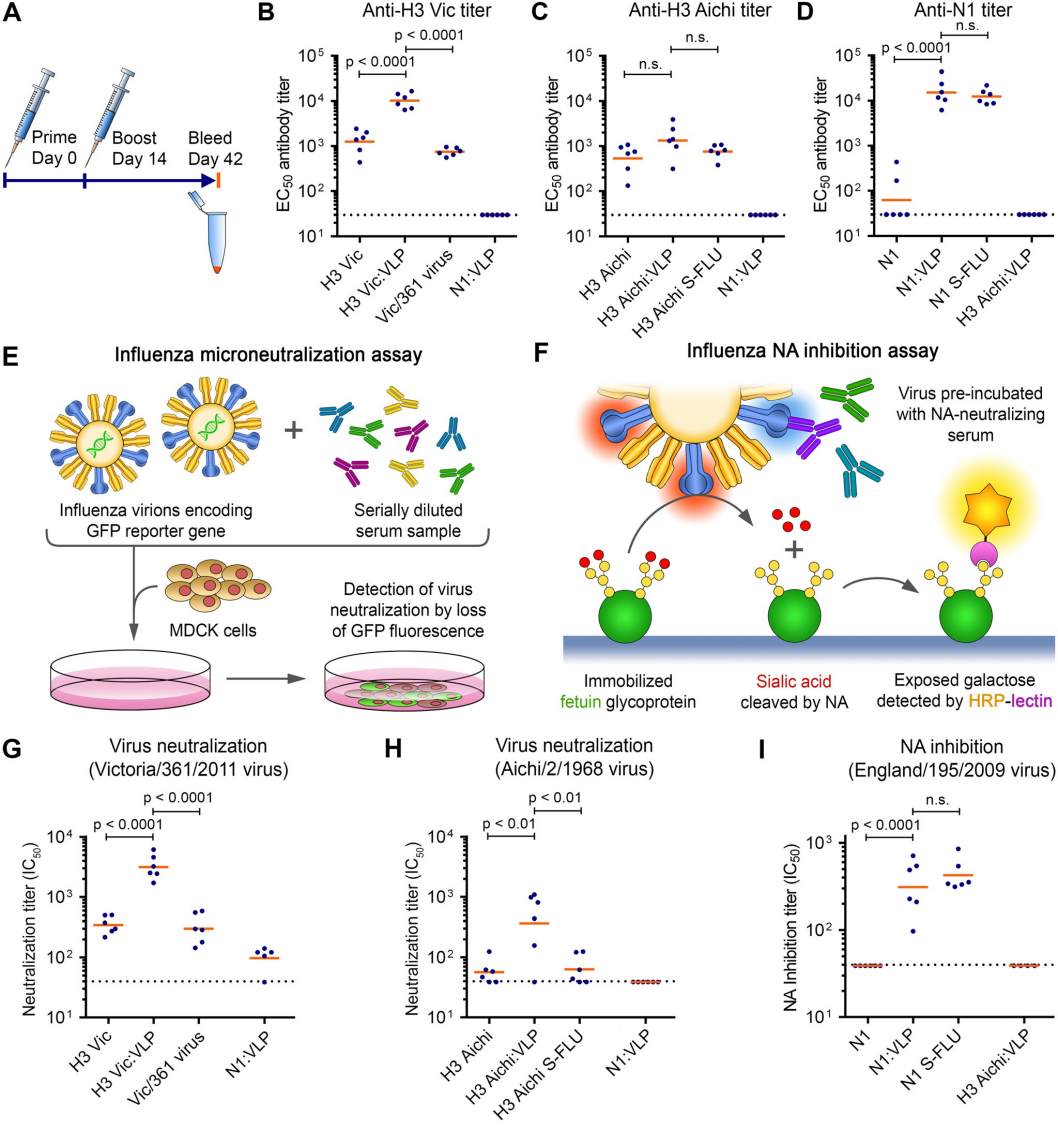
Display of trimeric or tetrameric influenza antigens on SpyCatcher003‐mi3 enhanced antibody titre. A) Immunization schedule. B) Antibody titer against H3 Vic from mice primed and boosted with free H3 Vic, H3 Vic displayed on SpyCatcher003‐mi3 (H3 Vic:VLP), or Vic/361 influenza virus. Each dot represents one mouse and the mean is indicated in orange. *n*=6. Sera were assayed against plasma membrane‐bound H3 Vic. Serum from mice immunized with N1:VLP was a negative control. C) As for (B) with H3 Aichi. D) As for (B) with N1. Serum from immunizing with H3 Aichi:VLP was a negative control. E) Schematic of microneutralization assay. Neutralization potency is quantified through the decrease in GFP in cells incubated with virus pre‐mixed with serum. F) Schematic of NA inhibition assay. Inhibition of NA catalytic activity is quantified through decrease of lectin‐HRP binding to immobilized fetuin in the presence of NA‐neutralizing antibodies. G) VLP‐display enhanced virus‐neutralizing activity from H3 Vic immunization. Microneutralization assay on serum following each immunization route, tested against A/Victoria/361/2011 influenza. Serum from immunizing with N1:VLP was a negative control. *n*=6, except N1:VLP *n*=5. H) Microneutralization assay as in (G) but for H3 Aichi and A/Aichi/2/1968 virus. I) NA inhibition assay for mice immunized with free N1, N1 displayed on VLP, or matching S‐FLU. Serum from immunizing with H3 Aichi:VLP was a negative control. *n*=6, except H3 Aichi:VLP *n*=4. Dashed lines indicate limit of detection (1:40 dilution). Significance was analyzed using log_10_‐transformed data and one‐way ANOVA with Bonferroni correction. n.s.=not significant.

Having confirmed facile decoration of trimeric or tetrameric influenza antigens on our VLP platform, we investigated the magnitude and quality of immune responses. We validated efficient VLP coupling of H3 Vic, H3 Aichi or N1 by SDS‐PAGE (Figure S7A). Mice were immunized intramuscularly with 0.1 μg antigen doses using a homologous prime‐boost regimen, along with AddaVax adjuvant (Figure 5 A). Boosting was performed 2 weeks after prime, with blood sampling 4 weeks after the boost. We compared immunization with the same antigen dose of non‐conjugated antigens or with influenza virion controls. For H3 Vic, wild‐type Victoria/361/2011 virus (termed Vic/361) was the control. For H3 Aichi and N1, single‐cycle influenza viruses (S‐FLU) pseudotyped with matching HA or NA were controls. S‐FLU is a promising viral vector vaccine: S‐FLU virions infect host cells but lack a gene to express more HA, so are incapable of productive viral infection.^[32]^ We analyzed antibody titers using a cell‐based assay. Serum samples are incubated with cells expressing surface H3 or N1. Then cell‐bound antibodies are detected using HRP‐conjugated antibody. Both H3 Vic and N1, when conjugated to SpyCatcher003‐mi3, induced higher median antibody titers than free antigen (7‐fold for H3 Vic, 490‐fold for N1, Figure 5 B–D). VLP‐display of H3 Aichi slightly increased the titer but this difference was not statistically significant (Figure 5 C). H3 Vic displayed on the VLP induced 14‐fold higher median titers than wild‐type virus, while both H3 Aichi:VLP and N1:VLP induced titers similar to S‐FLU (Figure 5 B–D).

As an alternative method for analyzing titer, we used indirect ELISA with antigens coated on a microtiter plate. The results largely matched the cell‐based assay, with both H3 Vic:VLP and N1:VLP giving significantly higher titers than free antigens (Figure S7B–D). In contrast to the cell‐based assay, in indirect ELISA all tested viral vectors had titers significantly lower than what was induced by VLP‐displayed antigens (Figure S7B–D).

Beyond the amount of antibodies, it is important to evaluate antibody efficacy. We studied the functionality in a virus microneutralization assay for HA‐specific antibodies (Figure 5 E).^[32]^ SpyCatcher003‐mi3 display of H3 variants improved the median neutralization titers by 8.5–12 fold compared to free antigen or virus‐based controls (Figure 5 G,H). To confirm specificity of neutralizing antibodies, we assayed the sera against S‐FLU pseudotyped with H3 Vic or H3 Aichi, giving similar results (Figure S7E,F).

The potency of NA‐specific antibodies in inhibition of NA catalysis was studied using an enzyme‐linked lectin assay (ELLA) (Figure 5 F).^[33]^ NA inhibition is an independent correlate of protection against influenza in clinical trials.^[31]^ VLP‐conjugated N1 showed potent NA inhibition, while none of the samples from immunization with free N1 showed detectable inhibition (Figure 5 I). Similar to N1:VLP, A/England/195/09 S‐FLU induced antibodies with potent inhibition of NA (Figure 5 I).

To investigate if antigen VLP‐display increased the functionality of the induced antibodies, we calculated IC_50_/EC_50_ ratios. Only samples with above‐threshold signal in both assays were included. VLP‐display of H3 Vic slightly increased the mean IC_50_/EC_50_ ratio compared to free antigen, but this difference was not statistically significant (Figure S7G). In contrast, VLP‐display of H3 Aichi significantly increased IC_50_/EC_50_, indicating improved antibody functionality (Figure S7 H). It was not possible to calculate IC_50_/EC_50_ for free N1, because IC_50_ was below detection threshold. N1:VLP and the matching S‐FLU gave similar IC_50_/EC_50_, indicating similar antibody functionality (Figure S7I). Taken together, the display of antigens on the SpyCatcher003‐mi3 platform not only increased antibody titers but can also improve antibody functionality.

## Discussion

Nature is able to fabricate atomically precise megadalton assemblies, which usually depend on multiple weak interactions evolved over numerous generations.^[3]^ Stable synthetic assemblies on this size‐scale may benefit from modular covalent conjugations, so it is important to advance their scope in nanoassembly. We have shown that modular amidation through SpyCatcher003 enables nanoassembly of diverse cyclic protein symmetries on a dodecahedral 60‐mer nanoparticle scaffold. Such generality is an important step towards the development of a broadly applicable platform to enhance the speed and efficiency of vaccine development.

Recombinant proteins, especially those from pathogens, often have marginal stability.[^6^, ^7^, ^8^] Therefore, linking many copies of such proteins on a single nanoparticle faces challenges from misfolding, aggregation or slow reaction near a crowded surface.^[9]^ We observed that SpyCatcher003‐mi3 allows display of antigens with diverse sizes and multimeric structures. Coupled particles were monodisperse by DLS, while VLP conjugation had a small thermostabilizing effect on coupled antigens. Despite the D2 symmetry of streptavidin and approximately antiparallel orientation of the NDV HN protein dimer, both were successfully decorated on SpyCatcher003‐mi3 while retaining high solubility. Similarly, all tested influenza antigens exhibited good solubility after VLP decoration. In contrast, particles decorated with the D2 symmetric β‐galactosidase almost completely aggregated. These data suggest that with large antigens, dihedral symmetry of antigen subunits can lead to antigen‐mediated VLP crosslinking and aggregation. Cyclic symmetry orients the binding sites in a single direction and is favored for membrane‐anchored antigens.^[34]^ C3 is likely to be the most common symmetry among oligomeric antigens.[^6^, ^7^, ^8^] We did not consider antigens with cubic symmetry (e.g. icosahedral shells) or unbounded symmetry (e.g. helical filaments),^[34]^ since such proteins should already have sufficient multivalency to promote strong immune response.^[8]^

Owing to the improved properties of SpyCatcher003, the SpyCatcher003‐mi3 platform exhibits high stability and activity. The platform can be expressed to high yields in *E. coli* and purified without affinity chromatography, through scalable and cost‐efficient ammonium sulfate precipitation and SEC. mi3 is based on an enzyme from a hyperthermophilic marine bacterium.[^14^, ^15^] Thus, existing human sero‐reactivity against the particle itself is unlikely. Here, we detected anti‐SpyTag/SpyCatcher antibodies in almost all human serum samples, while anti‐SpyTag003/SpyCatcher003 had 5‐fold decreased response. This reduced response is understandable, considering the 18 amino acid substitutions in the 003 pair.^[16]^ How pre‐existing anti‐platform antibodies shape vaccine response has been investigated in several studies: for Hepatitis B surface antigen (HBsAg), existing responses to the VLP or a homologous prime‐boost regimen do not impair later antigen‐specific immune response.[^35^, ^36^] In contrast to VLPs, anti‐platform responses are problematic where cell infection is required for vaccine function, for example, adenoviral vectors.^[37]^ Pre‐existing human antibodies against the orthogonal SnoopTag/SnoopCatcher pair were detected in only 25 % of people. Therefore, SnoopTag‐mediated assembly in heterologous prime‐boost immunization is an option, if repeated use of SpyCatcher003‐mi3 is ever problematic. Tag/Catcher conjugation has been applied for decoration of CAR‐T cells and in vivo imaging,^[10]^ where the consequences of pre‐existing immune responses for clinical development are likely to be more important.

Immunization with influenza HA or NA displayed on Spy‐VLPs gave superior antibody functionality compared to free antigen, which may be associated with both the multivalency and the orientation being similar to natural virions. Previous strategies to create HA‐containing nanoassemblies include budding of influenza VLPs from cells, as well as genetic fusion of HA to protein cages.^[38]^ While this work was in progress, the original SpyTag/SpyCatcher was used for multimerization of a truncated and non‐glycosylated monomeric HA head.^[39]^ Modular nanoassembly of HA by SpyCatcher003‐mi3 allows VLP stockpiling, which may help timely response to new influenza pandemics and the yearly variation of circulating influenza strains.[^28^, ^29^, ^30^] Modular vaccine assembly and tunable antigen density may also help to ensure high immunogenicity and access to the broadly neutralizing epitopes in the HA stem domain.^[6]^ In contrast to HA, few studies have investigated display of NA on protein VLPs.[^40^, ^41^]

## Conclusion

Spy‐VLP display may facilitate the next generation of recombinant influenza vaccines, inducing potent activity against both HA and NA. Together, we demonstrate that the SpyCatcher003‐mi3 platform is well suited for the display of complex antigens of diverse symmetry. Beyond vaccines, Spy‐based nanoassembly has enabled advances in catalysis, biomaterials and diagnostics, where applicability to different symmetry states may be similarly beneficial.[^42^, ^43^]

## Conflict of interest

M.H. is an inventor on a patent regarding spontaneous amide bond formation (EP2534484) and a patent application regarding SpyTag003:SpyCatcher003 (UK Intellectual Property Office 1706430.4). M.H. and S.J.D. are SpyBiotech co‐founders, shareholders and consultants. A.R.T. is an inventor on a patent relating to S‐FLU (EP2758525A2).

## Supporting information

As a service to our authors and readers, this journal provides supporting information supplied by the authors. Such materials are peer reviewed and may be re‐organized for online delivery, but are not copy‐edited or typeset. Technical support issues arising from supporting information (other than missing files) should be addressed to the authors.

Supplementary
