# Supplementary material for: Overcoming Symmetry Mismatch in Vaccine Nanoassembly through Spontaneous Amidation
Source: Angew Chem Int Ed Engl. 2020 Oct 27;60(1):321–30. doi: 10.1002/anie.202009663 (PMC7821241; doi:10.1002/anie.202009663)
Supplement: Supplementary file 1 — Supplementary [file ANIE-60-321-s001.pdf]

Angewandte Chemie
Eine Zeitschrift der Gesellschaft Deutscher Chemiker

Supporting Information

Overcoming Symmetry Mismatch in Vaccine Nanoassembly through Spontaneous Amidation

Rolle Rahikainen+, Pramila Rijal+, Tiong Kit Tan+, Hung-Jen Wu, Anne-Marie C. Andersson, Jordan R. Barrett, Thomas A. Bowden, Simon J. Draper, Alain R. Townsend, and Mark Howarth**

anie_202009663_sm_miscellaneous_information.pdf

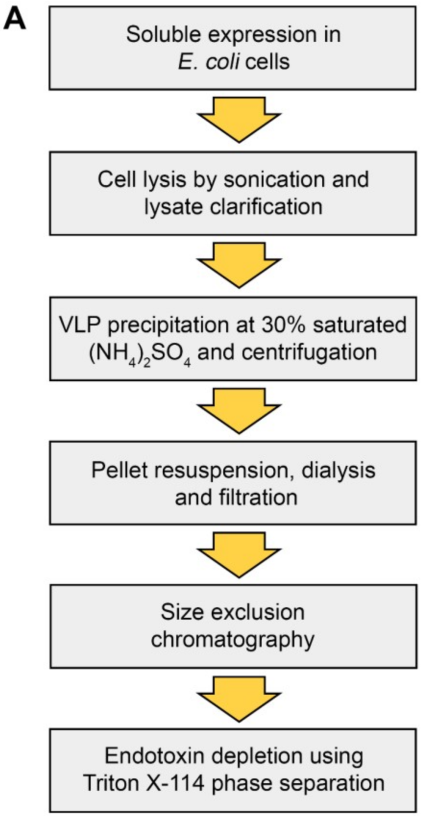

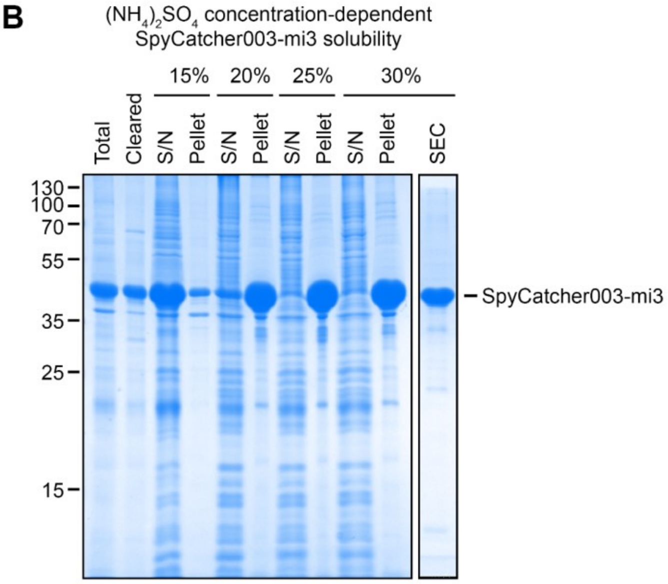

C

Purification step	Total protein (mg)	Target protein (mg)	Purity (%)	Relative yield (%)	Yield per L culture (mg)
Cleared lysate	72	59	82	100	234
$(NH_4)_2SO_4$ Precipitation	26	24	92	41	96
SEC	25	24	95	40	95

Figure S1. Affinity-independent Spy-VLP purification. (A) Process diagram for scalable SpyCatcher003-mi3 purification using $(NH_4)_2SO_4$ precipitation and size exclusion chromatography. (B) Nanoparticle purification using $(NH_4)_2SO_4$. *E. coli* total lysate was centrifuged and filtered to give cleared lysate and incubated with increasing ammonium sulfate concentrations, as analyzed by SDS-PAGE with Coomassie staining. Supernatant (S/N) and pellet were compared and the 30% resuspended fraction was further purified by SEC. (C) Purification table, showing the yield and purity of a representative SpyCatcher003-mi3 purification using $(NH_4)_2SO_4$ and SEC.

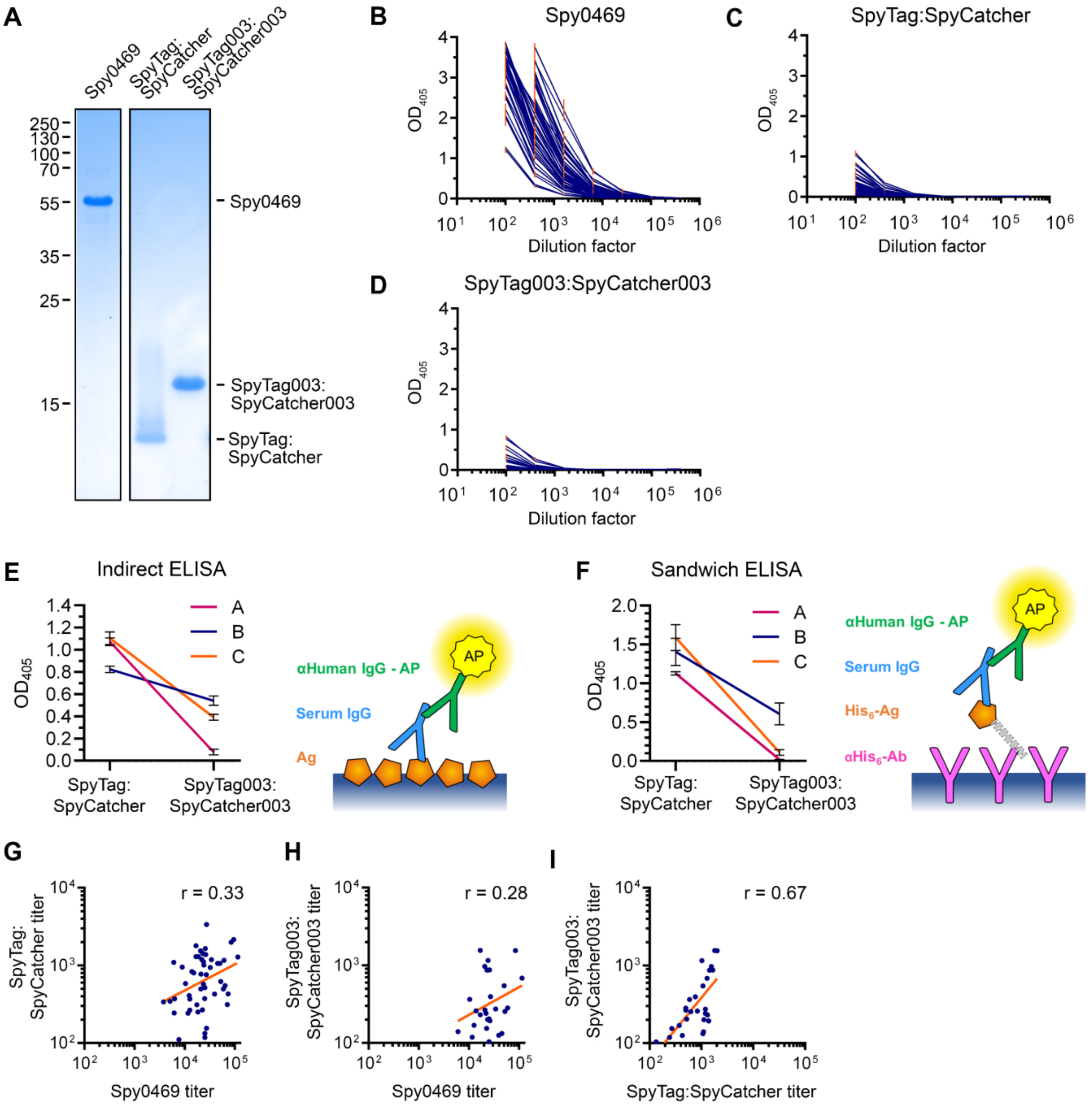

Figure S2. Testing of human sero-reactivity to SpyTag/SpyCatcher. (A) Antigen validation. Samples of antigens for pre-immunity analysis were resolved on non-reducing SDS-PAGE with Coomassie staining. As previously, after reaction with a short peptide, SpyCatcher showed gel-matrix interactions and migrated anomalously.[46] (B-D) Titration curves for antibody responses to each antigen. Each curve represents mean of two replicates for one person with error bars ± 1 s.d. presented as orange lines. In (B), data-points reaching detector saturation were omitted. (E-F) Comparison of indirect ELISA and sandwich ELISA for testing pre-existing antibodies to SpyTag/SpyCatcher or SpyTag003/SpyCatcher003 from three selected serum samples. Mean ± 1 s.d., n = 2. Schematics of each assay are shown alongside. (G-I) Correlation analysis for pre-existing antibody titers against Tag/Catcher pairs and *S. pyogenes* control protein Spy0469. Samples with responses below the detection threshold for either protein were omitted. A blue dot represents one person, with the best-fit linear regression line in orange. Pearson correlation coefficient for $\log_{10}$-transformed data is shown; for (G) n = 51, for (H-I) n = 27.

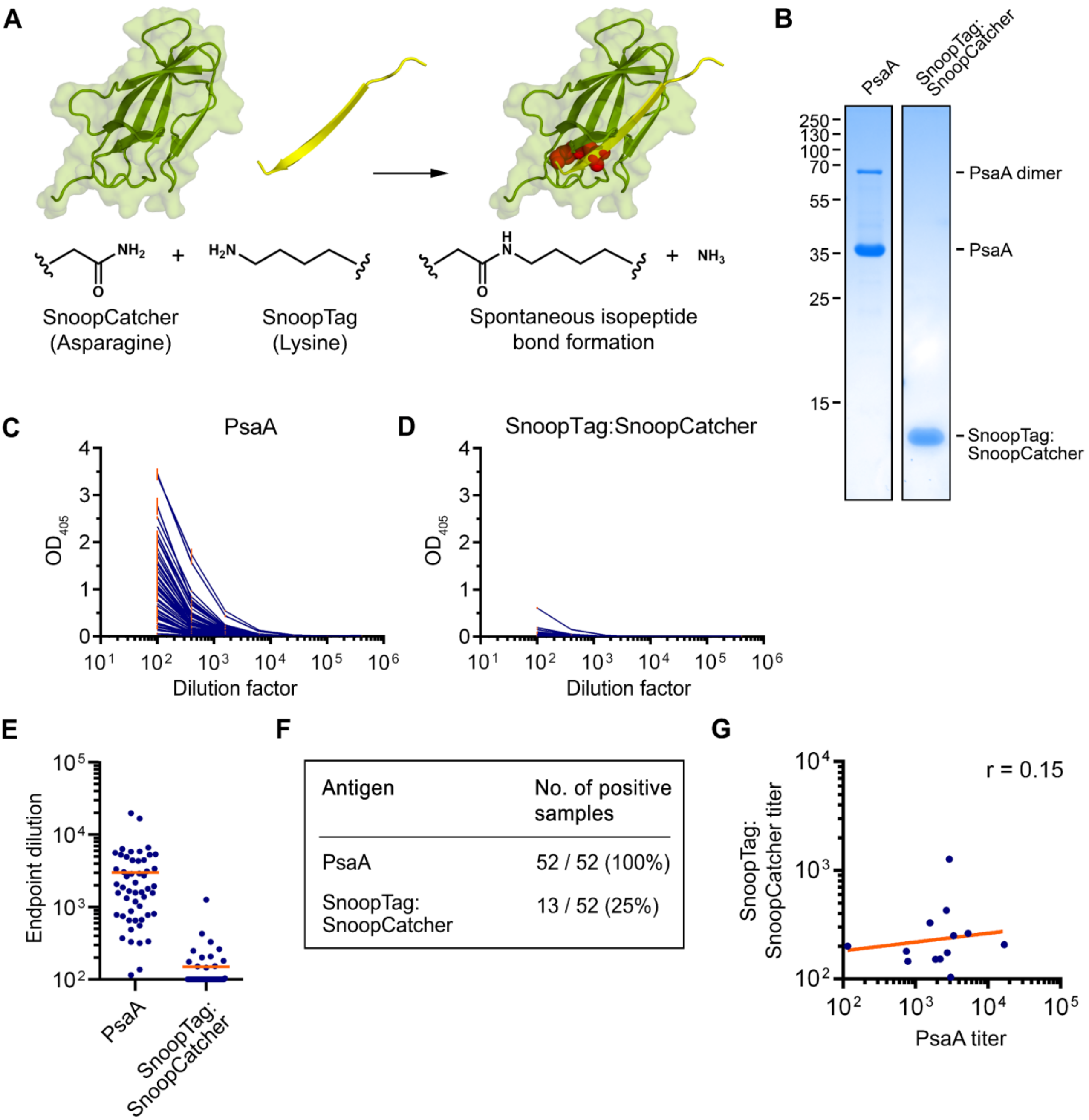

Figure S3. Testing of human sero-reactivity to SnoopTag/SnoopCatcher. (A) Schematic for bio-orthogonal SnoopTag/SnoopCatcher pair. An asparagine on SnoopCatcher (green) spontaneously forms an amide bond with a lysine on SnoopTag (yellow), based on PDB ID: 2WW8. Atoms forming the isopeptide bond are colored as red spheres. (B) Antigen validation. Samples of antigens for pre-immunity analysis were resolved on non-reducing SDS-PAGE with Coomassie staining. A fraction of PsaA ran as a disulfide-linked dimer. (C-D) Titration curves for antibody responses to each antigen. Each curve represents mean of two replicates for one person with error bars ± 1 s.d. presented as orange lines. (E) Pre-existing response to *S. pneumoniae* antigens. Antibody titer of serum from 52 healthy UK adults against the positive control PsaA or SnoopTag/SnoopCatcher. Each person is shown as a dot, with the mean in orange. (F) Summary of immune responses in (E). (G) Correlation analysis for pre-existing antibody titers against SnoopTag/SnoopCatcher and *S. pneumoniae* control protein PsaA. Samples with responses below the detection threshold for either protein were omitted. A blue dot represents one person, with the best-fit linear regression line in orange. Pearson correlation coefficient for $\log_{10}$-transformed data is shown. n = 13.

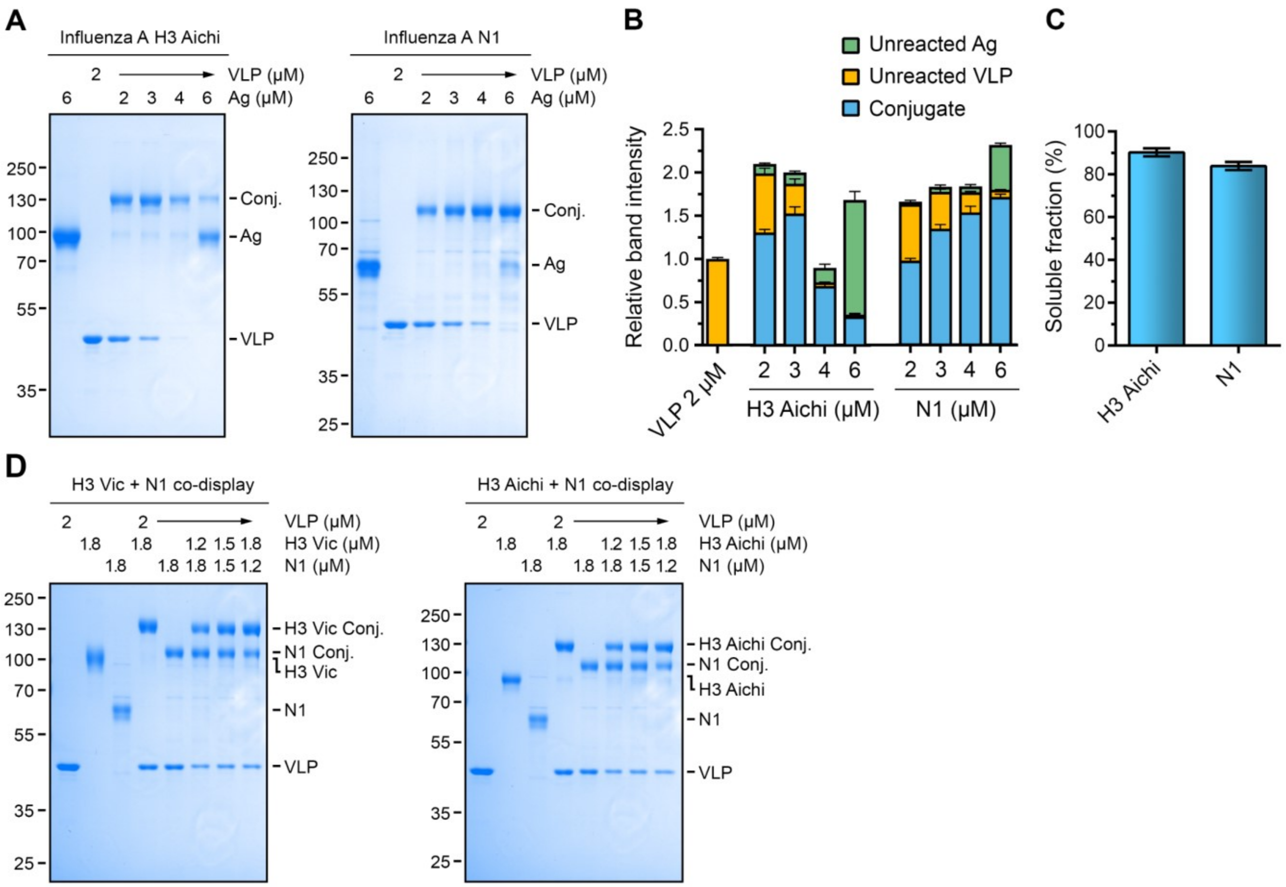

Figure S4. VLP coupling to Influenza antigens. (A) Coupling of H3 Aichi and N1 with SpyCatcher003-mi3 at different molar ratios. All samples were incubated for 16 h at 4 °C, insoluble aggregates removed by centrifugation, and supernatant loaded on reducing SDS-PAGE before Coomassie staining. Each gel is representative of three replicates. High excess of H3 Aichi led to decreased solubility of decorated VLPs. (B) Quantification of VLP:antigen reactions. The intensity of each band from gel densitometry was divided by the intensity of the band of VLP alone at 2 μM. Bars represent mean + 1 s.d., n = 3. (C) Soluble fraction of antigens conjugated at 1:1 molar ratio. Bars indicate mean ± 1 s.d., n = 3. (D) Co-display of H3 Victoria or H3 Aichi with N1 on a single particle. Antigens were mixed with the VLP at the indicated molar concentrations and incubated for 16 h at 4 °C, insoluble aggregates removed by centrifugation, and supernatant loaded on reducing SDS-PAGE before Coomassie staining.

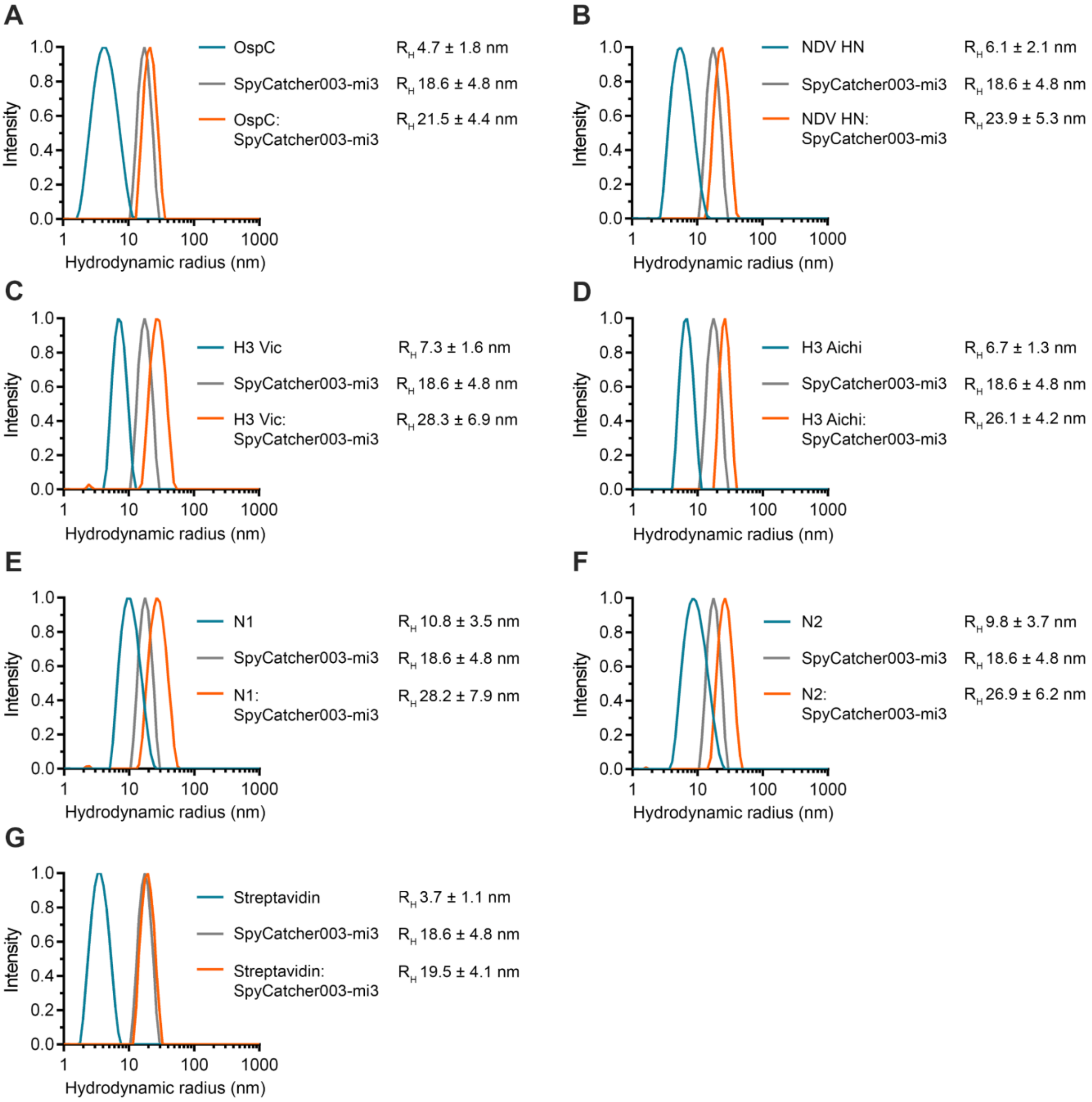

Figure S5. Hydrodynamic size of antigens before and after VLP coupling. (A-G) DLS for each multimeric antigen either alone or after conjugation with SpyCatcher003-mi3 at 1:1 molar ratio. For each antigen, mean hydrodynamic radius (R_H) and s.d. of the particle size distribution of a representative experiment are presented. n = 2-3 independent experiments for each sample.

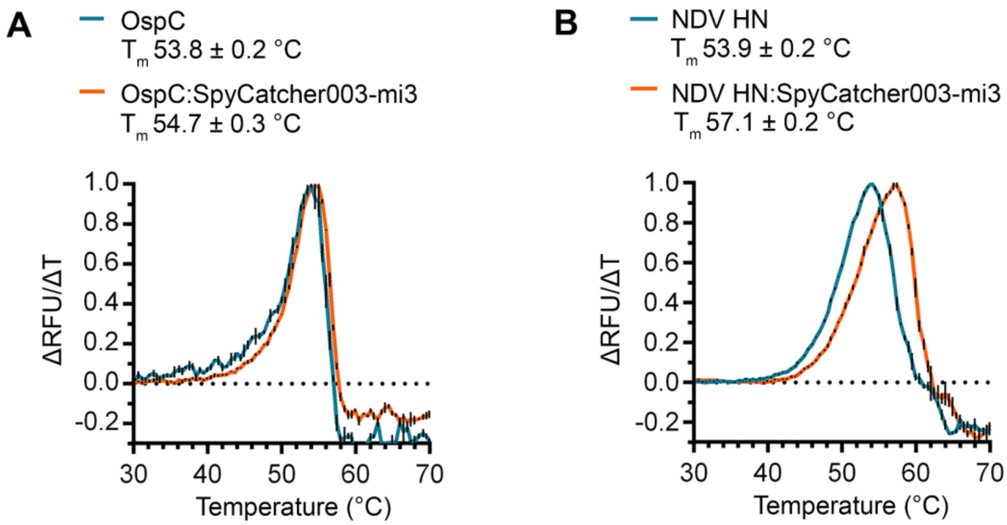

Figure S6. Thermofluor assay of antigens after VLP coupling. (A-B) Thermofluor assay for dimeric OspC and NDV HN antigens as free proteins or after VLP decoration. Graphs present the 1st derivative fluorescence signal (ΔRFU/ΔT) of three replicates. T_m values are mean ± 1 s.d., n = 3. Error bars, shown in black on the curves, are mean ± 1 s.d., n = 3.

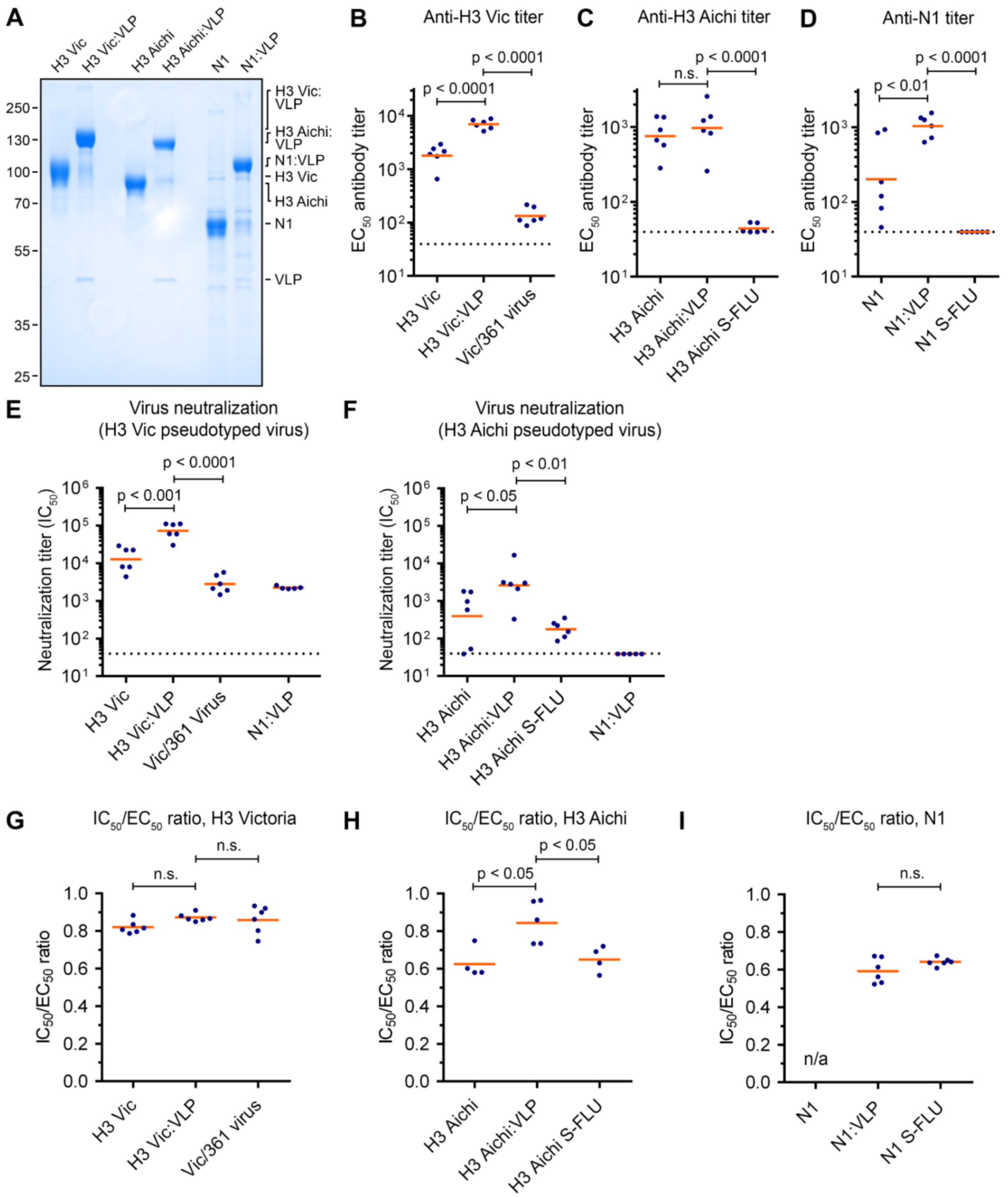

Figure S7. Immunization with oligomeric Influenza antigens. (A) Immunogens were resolved on SDS-PAGE with Coomassie staining to confirm successful coupling for 2 µM VLP with 3 µM H3 Vic, 3 µM H3 Aichi or 4 µM N1. (B-D) Indirect ELISA EC_{50} titers for antibodies from H3 Vic, H3 Aichi and N1 immunizations. Each dot represents one mouse and the mean titer is indicated with an orange line. (E) Microneutralization assay to test antibody blockade of S-FLU infection of MDCK cells. Neutralization titer for the indicated S-FLU was determined for serum from mice immunized with H3 Vic, H3 Vic coupled to SpyCatcher003-mi3, or the matching S-FLU. Serum from mice immunized with N1:VLP was used as a negative control. n = 6 for all groups except N1:VLP, where n = 5. Dashed lines indicate the lowest dilution factor (1:40) used in all assays. (F) As for (E) but with H3 Aichi. (G) IC_{50}/EC_{50} ratios for sera from H3 Vic immunizations. IC_{50}/EC_{50} ratios were calculated using log_{10}-transformed IC_{50} (microneutralization assay) and EC_{50} (cell-based assay) titers. n = 6 in each group. (H) As for (G) but with H3 Aichi. n = 4-5 for each group. (I) As for (G) but with N1 EC_{50} from ELLA. Below-threshold EC_{50} titers prevent IC_{50}/EC_{50} calculation for free recombinant N1. n = 6 in each group. In (B-I), statistical testing of log_{10}-transformed EC_{50} and IC_{50} titers and IC_{50}/EC_{50} ratios was done using one-way ANOVA with Bonferroni correction.

Table S1. Amino acid sequence of proteins expressed. SpyTag and SpyTag003 are indicated in bold. His$_6$ tag (HHHHHH) and C-Tag (EPEA) are indicated with gray shading. The stop codon is indicated with a hyphen. SpyCatcher, SpyCatcher003, SnoopCatcher, Spy0469 and PsaA were cleaved with TEV protease to remove the His$_6$-tag after purification. SUMO-SnoopTagJr was cleaved with SUMO protease Ulp1 to generate tagless SnoopTagJr. TEV protease and SUMO protease cleavage sites are indicated with a slash. SpyTag-RBD, NDV HN-SpyTag, H3 Vic-SpyTag003, H3 Aichi-SpyTag003, SpyTag-N1 and SpyTag-N2 are presented with N-terminal signal peptides. Proteins expressed in *E. coli* are shown with an N-terminal methionine. Table continued on the next page.

SpyCatcher-mi3
MGSSDSATHIKFSKRDEDGKELAGATMELRDSSGKTISTWISDGQVKDFYLYPGKYTFVETAAPDGYEVATAITFTVNEQGQVTVNGKATKGDAHI
GGSGGSGGSGGSMKMEELFKKHKIVAVLRANSVEEAKKKALAVFLGGVHLIEITFTVPDADTVIKELSFLKEMGAIIGAGTVTSVEQARKAVESGAEFI
VSPHLDEEISQFAKEKGVFYMPGVMTPTELVKAMKLGHTILKLFPGEVVGPQFVKAMKGPFPNVKFVPTGGVNLDNVCEWFKAGVLAVGVGSAL
VKGTPVEVAEKAKAFVEKIRGCTEGSGEPEA-

SpyCatcher003-mi3
MGSSVTTLSGLSGEQGPSGDMTTEEDSATHIKFSKRDEDGRELAGATMELRDSSGKTISTWISDGHVKDFYLYPGKYTFVETAAPDGYEVATPIEFTV
NEDGQVTVDGEATEGDAHTGGSGGSGGSGGSMKMEELFKKHKIVAVLRANSVEEAKKKALAVFLGGVHLIEITFTVPDADTVIKELSFLKEMGAIIG
AGTVTSVEQARKAVESGAEFIVSPHLDEEISQFAKEKGVFYMPGVMTPTELVKAMKLGHTILKLFPGEVVGPQFVKAMKGPFPNVKFVPTGGVNLD
NVCEWFKAGVLAVGVGSALVKGTPVEVAEKAKAFVEKIRGCTEGSGEPEA-

SpyCatcher
MSYYHHHHHHDYDIPTTENLYFQ/GAMVDTLSGLSSEQGQSGDMTIEEDSATHIKFSKRDEDGKELAGATMELRDSSGKTISTWISDGQVKDFYLY
PGKYTFVETAAPDGYEVATAITFTVNEQGQVTVNGKATKGDAHI-

SpyCatcher003
MSYYHHHHHHDYDIPTTENLYFQ/GAMVTTLSGLSGEQGPSGDMTTEEDSATHIKFSKRDEDGRELAGATMELRDSSGKTISTWISDGHVKDFYLY
PGKYTFVETAAPDGYEVATPIEFTVNEDGQVTVDGEATEGDAHTGSSGS-

SnoopCatcher
MGHHHHHHGSGENLYFQ/GSGKPLRGAVFSLQKQHPDYPDIYGAIDQNGTYQNVRTGEDGKLTFKNLSDGKYRLFENSEPAGYKPVQNKPIVAFQ
IVNGEVRDVTSIVPQDIPATYEFTNGKHYITNEPIPPK-

SUMO-SnoopTagJr
MGSWSHHHHHHGSDSEVNQEAKPEVKPEVKPETHINLKVSDGSSEIFFKIKKTTPLRRLMEAFAKRQGKEMDSLRFLYDGIRIQADQTPEDLDMED
NDIIEAHREQIGG/GKLGSIEFIKVNKGS-

Spy0469
MGHHHHHHGSGENLYFQ/GSGQAQEWTPRSVTEIKSELVLVDNVFTYTVKYGDTLSTIAEAMGIDVHVLGDINHIANIDLIFPDTILTANYNQHGQ
ATNLTVQAPASSPASVSHVPSSEPLPQASATSQPTVPMAPPATPSDVPTTPFASAKPDSSVTASSELTSSTNDVSTELSSESQKQPEVPQEAVPTPKA
AETTEVEPKTDISEAPTSANRPVPNESASEEVSSAAPAQAPAEKEETSAPAAQKAVADTTSVATSNGLSYAPNHAYNPMNAGLQPQTAAFKEEVAS
AFGITSFSGYRPGDPGDHGKGLAIDFMVPENSALGDQVAQYAIDHMAERGISYVIWKQRFYAPFASIYGPAYTWNPMPDRGSITENHYDHVHVSF
NA-

PsaA
MGHHHHHHGSGENLYFQ/GSGGSACASGKKDTTSGQKLKVVATNSIIADITKNIAGDKIDLHSIVPIGQDPHEYEPLPEDVKKTSEADLIFYNGINLET
GGNAWFTKLVENAKKTENKDYFAVSDGVDVIYLEGQNEKGKEDPHAWLNLENGIIFAKNIAKQLSAKDPNNKEFYEKNLKEYTDKLDKLDKESKDKF
NKIPAEKKLIVTSEGAFKYFSKAYGVPSAYIWEINTEEEGTPEQIKTLVEKLRQTKVPSLFVESSVDDRPMKTVSQDTNIPIYAQIFTDSIAEQGKEGDSY
YSMMKYNLDKIAEGLAK-

SpyTag-RBD
MNTQILVFALIAIIPTNADKIGSG**AHIVMVDAYKPTK**GSGGSGGSGTGNITNLCPFGEVFNATRFASVYAWNRKRISNCVADYSVLYNSASFSTFKCY
GVSPTKLNDLCFTNVYADSFVIRGDEVRQIAPGQTGKIADYNYKLPDDFTGCVIAWNSNNLDSKVGGNYNYLYRLFRKSNLKPFERDISTEIYQAGST
PCNGVEGFNCYFPLQSYGFQPTNGVGYQPYRVVVLSFELLHAPATVCGPKK-

SpyTag003-OspC
MGSSHHHHHHSSGLVPRGS**RGVPHIVMVDAYKRYK**GSGESGNNSGKDGNASANSADESVKGPNLTEISKKITESNAVVLAVKEVETLLASIDELAT
KAIGKKIGNNGLEANQSKNTSLLSGAYAISDLIAEKLNVLKNEELKEKIDTAKQSSTEFTNKLKSEHAVLGLDNLTDDNAQRAILKKHANKDKGAAELEK
LFKAVENLSKAAQDTLKNAVKELTSPIVAESPKKP-

Table S1 (continued)

NDV HN-SpyTag
MGILPSPGMPALLSLVSLLSVLLMGCVAETGSMGASTPSDLVGIPTRISRAEEKITSALGSNQDVVDRIYKQVALESPLALLNTETTIMNAITSLSYQIN
GAANNSGWGAPIHDPDFIGGIGKELIVDNASDVTSFYPSAFQEHLNFIPAPTTGSGCTRIPSFDMSATHYCYTHNVILSGCRDHSHSHQYLALGVLRT
TATGRIFFSTLRSISLDDTQNRKSCSVSATPLGCDMLCSKVTETEEEDYNSAVPTLMAHGRLGFDGQYHEKDLDVTTLFEDWVANYPGVGGGSFIDG
RVWFSVYGGLKPNSPSDTVQEGKYVIYKRYNDTCPDEQDYQIRMAKSSYKPGRFGGKRIQQAILSIKVSTSLGEDPVLTVPPNTVTLMGAEGRILTVG
TSHFLYQRGSSYFSPALLYPMTVSNKTATLHSPYTFNAFTRPGSIPCQASARCPNSCVTGVYTDPYPLIFYRNHTLRGVFGTMLDSEQARLNPASAVF
DSTSRSRITRVSSSSTKAAYTTSTCFKVVKTNKTYCLSIAEISNTLFGEFRIVPLLVEILKNDGVREARSGGSGGSGGSG**AHIVMVDAYKPTK**GSGGTKH
HHHHH-

H3 Vic-SpyTag003
MKTIIALSYILCLVFAQKLPGNDNSTATLCLGHHAVPNGTIVKTITNDQIEVTNATELVQNSSIGEICDSPHQILDGENCTLIDALLGDPQCDGFQNKK
WDLFVERSKAYSNCYPYDVPDYASLRSLVASSGTLEFNNESFNWTGVTQNGTSSACIRRSNNSFFSRLNWLTHLNFKYPALNVTMPNNEQFDKLYI
WGVHHPGTDKDQIFLYAQSSGRITVSTKRSQQAVIPNIGSRPRIRNIPSRISIYWTIVKPGDILLINSTGNLIAPRGYFKIRSGKSSIMRSDAPIGKCNSEC
ITPNGSIPNDKPFQNVNRITYGACPRYVKQSTLKLATGMRNVPEKQTRGIFGAIAGFIENGWEGMVDGWYGFRHQNSEGRGQAADLKSTQAAID
QINGKLNRLIGKTNEKFHQIEKEFSEVEGRIQDLEKYVEDTKIDLWSYNAELLVALENQHTIDLTDSEMNKLFEKTKKQLRENAEDMGNGCFKIYHKC
DNACIGSIRNGTYDHDVYRDEALNNRFQIKGVGSGSGSPGSGYIPEAPRDGQAYVRKDGEWVLLSTFLGGSGGSGGSG**RGVPHIVMVDAYKRYK**
GSHHHHHH-

H3 Aichi-SpyTag003
MKTIIALSYIFCLALGQDLPGNDNSTATLCLGHHAVPNGTLVKTITDDQIEVTNATELVQSSSTGKICNNPHRILDGIDCTLIDALLGDPHCDVFQNET
WDLFVERSKAFSNCYPYDVPDYASLRSLVASSGTLEFITEGFTWTGVTQNGGSNACKRGPGSGFFSRLNWLTKSGSTYPVLNVTMPNNDNFDKLYI
WGIHHPSTNQEQTSLYVQASGRVTVSTRRSQQTIIPNIGSRPWVRGLSSRISIYWTIVKPGDVLVINSNGNLIAPRGYFKMRTGKSSIMRSDAPIDTCI
SECITPNGSIPNDKPFQNVNKITYGACPKYVKQNTLKLATGMRNVPEKQTRGLFGAIAGFIENGWEGMIDGWYGFRHQNSEGTGQAADLKSTQAA
IDQINGKLNRVIEKTNEKFHQIEKEFSEVEGRIQDLEKYVEDTKIDLWSYNAELLVALENQHTIDLTDSEMNKLFEKTRRQLRENAEDMGNGCFKIYHK
CDNACIESIRNGTYDHDVYRDEALNNRFQIKGVGSGSGSPGSGYIPEAPRDGQAYVRKDGEWVLLSTFLGGSGGSGGSG**RGVPHIVMVDAYKRYK**
GSHHHHHH-

SpyTag-N1
MKANLLVLLCALAAADAADPHHHHHHSSSGSG**AHIVMVDAYKPTK**GSGGSGGSGDYSDLQRVKQELLEEVKKELQKVKEEIIEAFVQELRKRGSLV
PRGSPSRSISNTNFAAGQSVVSVKLAGNSSLCPVSGWAIYSKDNSVRIGSKGDVFVIREPFISCSPLECRTFFLTQGALLNDKHSNGTIKDRSPYRTLMS
CPIGEVPSPYNSRFESVAWSASACHDGINWLTIGISGPDNGAVAVLKYNGIITDTIKSWRNNILRTQESECACVNGSCFTVMTDGPSNGQASYKIFRI
EKGKIVKSVEMNAPNYHYEECSCYPDSSEITCVCRDNWHGSNRPWVSFNQNLEYQIGYICSGIFGDNPRPNDKTGSCGPVSSNGANGVKGFSFKYG
NGVWIGRTKSISSRNGFEMIWDPNGWTGTDNNFSIKQDIVGINEWSGYSGSFVQHPELTGLDCIRPCFWVELIRGRPKENTIWTSGSSISFCGVNSD
TVGWSWPDGAELPFTIDK-

SpyTag-N2
MKANLLVLLCALAAADAADPHHHHHHSSSGSG**AHIVMVDAYKPTK**GSGGSGGSGDYSDLQRVKQELLEEVKKELQKVKEEIIEAFVQELRKRGSLV
PRGSPSRSLTNTTIEKEICPKPAEYRNWSKPQCGITGFAPFSKDNSIRLSAGGDIWVTREPYVSCDPDKCYQFALGQGTTLNNVHSNNTVRDRTPYRT
LLMNELGVPFHLGTKQVCIAWSSSSCHDGKAWLHVCITGDDKNATASFIYNGRLVDSVVSWSKEILRTQESECVCINGTCTVVMTDGSASGKADTK
ILFIKEGKIVHTSTLSGSAQHVEECSCYPRYPGVRCVCRDNWKGSNRPIVDINIKDHSIVSSYVCSGLVGDTPRKTDSSSSSHCLDPNNEEGGHGVKG
WAFDDGNDVWMGRTINETSRLGYETFKVIEGWSNPKSKLQINRQVIVDRGDRSGYSGIFSVEGKSCINRCFYVELIRGRKEETEVLWTSNSIVVFCG
TSGTYGTGSWPDGADLNLMPI-

Dead streptavidin-SpyTag
MAEAGITGTWYAQLGDTFIVTAGADGALTGTYEAAVGNAESRYVLTGRYDSAPATDGSGTALGWTVAWKNNYRNAHSATTWSGQYVGGAEAR
INTQWLLTSGTTEANAWKSTLVGHDTFTKVKPSAASGSGSGG**AHIVMVDAYKPT**-

SpyTag003-β-galactosidase
MGSSHHHHHHSSGLVPRGS**RGVPHIVMVDAYKRYK**GSGESGMTMITDSLAVVLQRRDWENPGVTQLNRLAAHPPFASWRNSEEARTDRPSQ
QLRSLNGEWRFAWFPAPEAVPESWLECDLPEADTVVVPSNWQMHGYDAPIYTNVTYPITVNPPFVPTENPTGCYSLTFNVDESWLQEGQTRIIFD
GVNSAFHLWCNGRWVGYGQDSRLPSEFDLSAFLRAGENRLAVMVLRWSDGSYLEDQDMWRMSGIFRDVSLLHKPTTQISDFHVATRFNDDFSR
AVLEAEVQMCGELRDYLRVTVSLWQGETQVASGTAPFGGEIIDERGGYADRVTLRLNVENPKLWSAEIPNLYRAVVELHTADGTLIEAEACDVGFR
EVRIENGLLLLNGKPLLIRGVNRHEHHPLHGQVMDEQTMVQDILLMKQNNFNAVRCSHYPNHPLWYTLCDRYGLYVVDEANIETHGMVPMNRL
TDDPRWLPAMSERVTRMVQRDRNHPSVIIWSLGNESGHGANHDALYRWIKSVDPSRPVQYEGGGADTTATDIICPMYARVDEDQPFPAVPKWS
IKKWLSLPGETRPLILCEYAHAMGNSLGGFAKYWQAFRQYPRLQGGFVWDWVDQSLIKYDENGNPWSAYGGDFGDTPNDRQFCMNGLVFADR
TPHPALTEAKHQQQFFQFRLSGQTIEVTSEYLFRHSDNELLHWMVALDGKPLASGEVPLDVAPQGKQLIELPELPQPESAGQLWLTVRVVQPNATA
WSEAGHISAWQQWRLAENLSVTLPAASHAIPHLTTSEMDFCIELGNKRWQFNRQSGFLSQMWIGDKKQLLTPLRDQFTRAPLDNDIGVSEATRID
PNAWVERWKAAGHYQAEAALLQCTADTLADAVLITTAHAWQHQGKTLFISRKTYRIDGSGQMAITVDVEVASDTPHPARIGLNCQLAQVAERVN
WLGLGPQENYPDRLTAACFDRWDLPLSDMYTPYVFPSENGLRCGTRELNYGPHQWRGDFQFNISRYSQQQLMETSHRHLLHAEEGTWLNIDGF
HMGIGGDDSWSPSVSAEFQLSAGRYHYQLVWCQK-

METHODS

Expression constructs. All expression constructs were cloned using standard PCR methods and Gibson assembly. DNA fragments encoding antigens were ordered from Integrated DNA Technologies unless described otherwise. All inserts were authenticated using Sanger sequencing (Source BioScience). See Supplementary Table 1 for the amino acid sequences of all proteins expressed. pET28a-SpyCatcher-mi3 (GenBank MH425515, Addgene 112255) has been described[44]. pET28a-SpyCatcher003-mi3 (GenBank MT945417, Addgene 159995) was created by replacing SpyCatcher in pET28a-SpyCatcher-mi3 with SpyCatcher003[45]. pET28a-His_6-Spy0469 (GenBank MT945418, Addgene 159996) was created by inserting a synthetic fragment encoding *S. pyogenes* (NS5496 strain) Spy0469 amino acids 24-389 into pET28a. pET28a-His_6-PsaA (GenBank MT945419, Addgene 159997) was created by inserting a synthetic fragment encoding *S. pneumoniae* (GPSC5 strain) PsaA amino acids 19-309 into pET28a. pET28a-His_6-SpyCatcher and pET28a-His_6-SpyCatcher003 were created by inserting SpyCatcher (GenBank JQ478411)[46] or SpyCatcher003 (GenBank MN433887)[45] into pET28a. pET28a-His_6-SnoopCatcher was created by inserting SnoopCatcher (GenBank KU500646)[47] into pET28a. pET28a-His_6-SUMO-SnoopTagJr (GenBank MT945420) was created by inserting *Saccharomyces cerevisiae* Small Ubiquitin-like Modifier (SUMO) protein (GenBank NM001180818) and SnoopTagJr (KLGSIEFIKVNK)[48] into pET28a. pET28a-His_6-SpyTag003-OspC (GenBank MT945421, Addgene 159998) was created by inserting His_6 tag, GSGESG linker, SpyTag003 (RGVPHIVMVDAYKRYK)[45] and *B. burgdorferi* N40 strain OspC (GenBank JN969070) amino acids 20-209 with C129S substitution, into pET28a. The C129S substitution was made to prevent OspC aggregation as previously described[49]. pET28a-His_6-SpyTag003-β-galactosidase was a kind gift from Juha Huiskonen, University of Helsinki, and contains His_6 tag, GSGESG linker, SpyTag003 and *E. coli* β-galactosidase amino acids 1-1024 cloned into pET28a. For streptavidin, we used the pET21-DTag construct (streptavidin bearing SpyTag at its C-terminus, with mutations reducing biotin-binding affinity) (GenBank KJ906520, Addgene ID 59548), which has been described[50]. pcDNA3.1-H3Vic-SpyTag003-His_6 (GenBank MT945422) was created by inserting Influenza A/Victoria/361/2011 H3 (GenBank KJ942680) amino acids 1-521 (synthesized by GeneArt), GSGSGSPGS linker, T4 bacteriophage fibritin foldon domain, $(GSG)_3$ linker, SpyTag003 and His_6 tag into pcDNA3.1. pcDNA3.1-H3Aichi-SpyTag003-His_6 (GenBank MT945423) was created in the same way using Influenza A/Aichi/2/1968 (GenBank EF614251) H3 coding sequence. The expression constructs for both H3 variants lack the natural 5' and 3' untranslated regions (UTRs) and the transmembrane domain. pcDNA3.1-His_6-SpyTag-VASP-N1 (GenBank MT945424) was created by cloning Influenza A/PR/8/1934 H1 signal sequence, His_6 tag, SSSGSG linker, SpyTag (AHIVMVDAYKPTK), $(GSG)_3$ linker, human Vasodilator-stimulated phosphoprotein (VASP) tetramerization domain (GenBank NM003370.4) amino acids 338-375 and Influenza A/England/195/2009 N1 (GenBank ACR15621) amino acids 69-469 (synthesized by GeneArt) into pcDNA3.1. pcDNA3.1-His_6-SpyTag-VASP-N2 (GenBank MT945425) was created in the same way using Influenza A/Victoria/361/2011 N2 (GenBank KJ942682) amino acids 68-469. The expression constructs for both N1 and N2 lack the natural 5' and 3' UTRs and the transmembrane domain. pcDNA3.1-SpyTag-RBD (GenBank MT945427, Addgene 159999) was created by cloning Influenza A/Guangdong/2017 H7 signal sequence, SpyTag, $(GSG)_3$ linker and SARS-CoV-2 spike glycoprotein (GenBank NC045512) amino acids 340-538 (synthesized by GeneArt) into pcDNA3.1. pHL-sec-NDV-HN-SpyTag-His_6 (GenBank MT945426, Addgene 160000) has the following organization in the pHL-sec backbone: chicken receptor tyrosine phosphatase α signal sequence, Avian avulavirus Kansas strain HN protein (GenBank AF212323) amino acids 47-575, $(GGS)_3$ linker, SpyTag,

GSGGTK linker, His_6. Vectors for His_6-tagged SUMO protease Ulp1[51] and His_6-MBP-TEV protease[52] have been described.

Expression and purification of SpyCatcher003-mi3. SpyCatcher003-mi3 was expressed in *E. coli* BL21(DE3) RIPL cells (Agilent). Heat-shock transformed cells were plated on LB-Agar plates with 50 µg/mL kanamycin and incubated for 16 h at 37 °C. A single colony was picked into 10 mL starter culture of LB media with 50 µg/mL kanamycin, and incubated for 16 h at 37 °C with shaking at 200 rpm. The pre-culture was diluted 1:100 into 1 L LB supplemented with 50 µg/mL kanamycin and 0.8% (w/v) glucose and cultured at 37 °C, 200 rpm until OD_{600} = 0.6. Protein expression was induced with 420 µM isopropyl β-D-1-thiogalactopyranoside (IPTG) and incubation was continued at 22 °C, 200 rpm for 16 h. The cell pellet from 500 mL culture was resuspended in 20 mL 25 mM Tris-HCl, 300 mM NaCl, pH 8.5, supplemented with 0.1 mg/mL lysozyme, 1 mg/mL cOmplete mini EDTA-free protease inhibitor (Merck) and 1 mM phenylmethanesulfonyl fluoride (PMSF). Cell suspension was incubated at 22 °C for 30 min on a platform shaker and sonicated on ice 4 times for 60 s at 50% duty-cycle using an Ultrasonic Processor (Cole-Parmer) equipped with a microtip. Cell lysate was clarified by centrifugation at 35,000 g for 45 min at 4 °C. The supernatant was filtered through both 0.45 µm and 0.22 µm syringe filters (STARLAB) and 170 mg ammonium sulfate was added per mL of lysate. SpyCatcher003-mi3 particles were precipitated by incubating the lysate at 4 °C for 1 h while mixing at 100 rpm with a magnetic stirrer. Precipitated particles were pelleted by centrifugation at 30,000 g for 30 min at 4 °C. Supernatant was discarded and the pellet was resuspended into 8 mL 25 mM Tris-HCl, 150 mM NaCl, pH 8.5. Residual ammonium sulfate was removed by dialyzing for 16 h against 500-fold excess of the same buffer. Dialyzed SpyCatcher003-mi3 was centrifuged at 17,000 g for 30 min at 4 °C to pellet any insoluble material. The supernatant was filtered through a 0.22 µm syringe filter to further remove insoluble material. 2.5 mL was loaded into a HiPrep Sephacryl S-400 HR 16-600 SEC column (GE Healthcare) equilibrated with 25 mM Tris-HCl, 150 mM NaCl, pH 8.5 using an ÄKTA Pure 25 system (GE Healthcare). Proteins were separated at 1 mL/min while collecting 1 mL elution fractions. The fractions containing the purified particles were identified by SDS-PAGE, pooled, and concentrated using a Vivaspin 20 100 kDa M_W cut-off spin concentrator. Endotoxin was removed from the SpyCatcher003-mi3 samples using Triton X-114 phase separation as previously described[52]. Briefly, 1% (v/v) Triton X-114 was added to SpyCatcher003-mi3 samples and the solution was mixed by gentle pipetting. The samples were incubated on ice for 15 min or until Triton X-114 had completely dissolved. The tubes were transferred to a 37 °C water bath and incubated for 5 min. Micellar Triton X-114 was separated by centrifugation at 16,900 g for 5 min at 30 °C and the top phase was carefully separated into a fresh endotoxin-free microcentrifuge tube. Triton X-114 phase separation was repeated twice more. After the third round, residual Triton X-114 was removed by an additional incubation at 37 °C for 5 min, followed by centrifugation at 16,900 g for 10 min at 30 °C. The concentration of endotoxin-depleted particles was measured using bicinchoninic acid (BCA) assay (Pierce) and particles were stored at -80 °C. Estimated yield per L culture was calculated based on a purification using 250 mL of cell suspension. VLP sample purity was estimated using SDS-PAGE and gel densitometry.

Expression and purification of OspC and β-galactosidase. SpyTag003-OspC and SpyTag003-β-galactosidase were expressed in *E. coli* BL21(DE3) RIPL cells (Agilent). 5 mL overnight cultures were diluted into 1 L LB supplemented with 50 µg/mL kanamycin and 0.8% (w/v) glucose. Cultures were incubated at 37 °C, 200 rpm until OD_{600} = 0.5 and protein expression was induced with 840 µM IPTG for β-galactosidase or 420 µM IPTG for OspC. Incubation was continued for 3 h at 37 °C, 200 rpm for β-galactosidase and 3 h at 30 °C, 200

rpm for OspC. Cell pellets from 1 L cultures were resuspended into 20 mL (β-galactosidase) or 15 mL (OspC) of 50 mM Tris-HCl pH 7.8, 300 mM NaCl supplemented with 0.1 mg/mL lysozyme, 1 mg/mL cOmplete mini EDTA-free protease inhibitor and 1 mM PMSF. β-galactosidase lysis buffer was supplemented with 2 mM 2-mercaptoethanol and OspC lysis buffer with 10 mM imidazole. Cells were lysed by incubation for 1 h at 4 °C on a roller shaker, followed by sonication on ice 4 times for 60 s each at 50% duty-cycle using an Ultrasonic Processor (Cole-Parmer) equipped with a microtip. Cell debris was pelleted by centrifugation at 35,000 g for 45 min at 4 °C and the supernatant was mixed with 1 mL Ni-NTA sepharose (Qiagen) equilibrated with the lysis buffer. After 30 min incubation at 4 °C on a roller shaker, the mixtures were added to gravity-driven Econo-Pac polypropylene columns (Bio-Rad) and resin was washed with 15 column volumes (CV) of lysis buffer, followed by 15 CV of lysis buffer with 10 mM imidazole and then 5 CV of lysis buffer with 30 mM imidazole. Bound proteins were eluted by 200 mM imidazole in the lysis buffer and imidazole was removed by dialysis for 16 h using 3.5 kDa Spectra/Por-3 dialysis membrane (Spectrum Chemical). SpyTag003-β-galactosidase was dialyzed once against 25 mM Tris-HCl pH 7.5, 150 mM NaCl, 5 mM EDTA, 2 mM tris(2-carboxyethyl)phosphine (TCEP), followed by dialyzing twice against 25 mM Tris-HCl pH 7.5, 150 mM NaCl, 2 mM $MgCl_2$, 2 mM $CaCl_2$, 2 mM TCEP. SpyTag003-OspC was dialyzed once against 25 mM Tris-HCl pH 7.5, 150 mM NaCl, 5 mM EDTA and twice against 25 mM Tris-HCl pH 7.5, 150 mM NaCl. Dialyzed proteins were concentrated using VivaSpin spin concentrators with 100 kDa cut-off for β-galactosidase and 30 kDa cut-off for OspC. Concentrated proteins were further purified using an ÄKTA Pure 25 (GE Life Sciences) and size exclusion columns. For both proteins, the last dialysis buffer was used as the moving phase. SpyTag003-β-galactosidase was purified using Superose 6 Increase 10-300 column (GE Life Sciences) at 0.75 mL/min. SpyTag003-OspC was purified using a HiLoad Superdex 75pg 16-600 column (GE Life Sciences) at 1 mL/min. Purified proteins were concentrated using the same spin concentrators as before and stored at -80 °C. The final yields per L of culture medium were 3.4 mg for SpyTag003-OspC and 1.5 mg for SpyTag003-β-galactosidase.

Expression and purification of Streptavidin-SpyTag fusion. Dead Streptavidin-SpyTag homotetramer was expressed in *E. coli* BL21(DE3) RIPL cells (Agilent) and purified from inclusion bodies as previously described[50]. Briefly, 15 mL overnight cultures were diluted into 1 L LB supplemented with 100 µg/mL ampicillin and 0.8% (w/v) glucose. Cultures were incubated at 37 °C, 200 rpm until OD_{600} = 0.6 and protein expression was induced with 420 µM IPTG. Incubation was continued for 4 h at 37 °C, 200 rpm. Cell pellets from 1 L cultures were resuspended into 15 mL PBS (137 mM NaCl, 2.7 mM KCl, 10 mM Na_2HPO_4, 1.7 mM KH_2PO_4, pH 7.4), supplemented with 1% (v/v) Triton X-100, 0.1 mg/mL lysozyme, 1 mg/mL cOmplete mini EDTA-free protease inhibitor, 1 mM PMSF and 10 mM EDTA. Cells were lysed by incubation for 30 min at 22 °C on a platform shaker, followed by sonication on ice 3 times for 60 s each at 50% duty-cycle using an Ultrasonic Processor (Cole-Parmer) equipped with a microtip. Insoluble material was pelleted by centrifugation at 27,000 g for 20 min at 4 °C, the supernatant discarded and the pellet containing streptavidin inclusion bodies carefully resuspended in 10 mL PBS supplemented with 1% (v/v) Triton X-100, 1 mg/mL cOmplete mini EDTA-free protease inhibitor, 1 mM PMSF and 10 mM EDTA. The mixture was diluted with additional 20 mL of the same buffer and centrifuged at 27,000 g for 20 min at 4 °C. The inclusion bodies were washed three more times, each time resuspending the pellet into 30 mL PBS supplemented with 10 mM EDTA. The final pellet was resuspended into 6 mL 6 M guanidinium hydrochloride pH 1.5 and any remaining solid material pelleted by centrifugation at 27,000 g for 30 min at 4 °C. For streptavidin refolding, the supernatant was left to slowly drip through a 10 µL pipet tip into 250 mL ice cold PBS with 10 mM EDTA mixed at high

speed on a magnetic stirrer. The mixing speed was lowered to 150 rpm and the solution left to mix for 16 h at 4 °C. Any insoluble material was removed by filtration though cellulose filter paper (Whatman) and the streptavidin tetramers in the flow-through were precipitated with 150 g ammonium sulfate per 250 mL PBS (60% of the saturation point at 4 °C). The mixture was incubated for 2 h with gentle mixing and the precipitate was pelleted by centrifugation at 20,000 g for 30 min at 4 °C. The supernatant was discarded and the pellet dissolved into 15 mL 25 mM Tris-HCl, 150 mM NaCl, 5 mM EDTA pH 8.0 and dialyzed twice against the same buffer. Any precipitated material was pelleted by centrifugation at 4,500 g for 10 min at 4 °C. The refolded streptavidin was further purified using 2-iminobiotin affinity chromatography. The protein sample was added to a 1 mL column of 2-iminobiotin agarose resin (Affiland) pre-equilibrated with 50 mM sodium borate, 500 mM NaCl, pH 10.0 (binding buffer). The column was left to empty by gravity flow and the resin was washed twice with 15 CV of the binding buffer. Resin-bound streptavidin was eluted with 15 CV of 20 mM NaH_2PO_4 pH 2.0, collecting 1 mL fractions into microcentrifuge tubes containing 100 µL 1 M Tris-HCl, pH 8.0. Pooled elution fractions were dialyzed against PBS and concentrated using a Vivaspin 20 30 kDa spin filter.

Expression and purification of Influenza antigens and NDV HN. Influenza antigens and NDV HN were expressed in Expi293F cells (Thermo Fisher) following manufacturer's recommendations. Briefly, Expi293F cells were cultured in a humidified Multitron Cell incubator (Infors HT) at 37 °C with 8% (v/v) CO_2, rotating at 125 rpm, for at least 3 passages before transient transfection. Cells were transfected at 2.5×10^6 cells/mL using 2.7 µL ExpiFectamine 293 (Thermo Fisher) and 1 µg endotoxin-free plasmid DNA per 1 mL of cell suspension. All proteins were expressed at 60 mL total culture volume in vented 250 mL shake flasks (Corning). Transfection enhancers 1 and 2 (Thermo Fisher) were added 18 h post-transfection and culture continued until cell viability dropped under 40-60% (4-6 days post-transfection). Proteins were purified from cell culture supernatants using Ni-NTA-sepharose and SEC. Briefly, cell culture supernatants were clarified by centrifugation at 3,500 g for 5 min at 22 °C, followed by filtration through a 0.22 µm syringe filter. Cleared supernatants were mixed with one quarter supernatant volume of 50 mM Tris-HCl pH 8.0, 300 mM NaCl. For H3, N1 and N2, 10 mM imidazole was added to the supernatants and the pH adjusted to 8.0 using 0.1 M NaOH. The supernatants were mixed with 0.75-1.5 mL Ni-NTA-sepharose (Qiagen) pre-equilibrated with 50 mM Tris-HCl pH 8.0, 300 mM NaCl and the mixtures were incubated on a roller shaker for 1 h at 4 °C. The mixtures were loaded into polypropylene columns and washed with 10 CV of 10 mM imidazole in 50 mM Tris-HCl pH 8.0, 300 mM NaCl and then 5 CV of 30 mM imidazole in 50 mM Tris-HCl pH 8.0, 300 mM NaCl. Proteins were eluted with 200 mM imidazole in 50 mM Tris-HCl pH 8.0, 300 mM NaCl. H3 Vic, H3 Aichi, N1 and N2 were dialyzed against 25 mM Tris-HCl pH 8.0, 150 mM NaCl, 10 mM EDTA. NDV HN was dialyzed against 25 mM Tris-HCl pH 8.0, 150 mM NaCl. All proteins were concentrated with VivaSpin 20 30 kDa spin filters and further purified using SEC. H3 Vic and H3 Aichi were purified using Superose 6 increase 10-300 column (GE Life Sciences) equilibrated with 25 mM Tris-HCl pH 8.0, 150 mM NaCl at 0.75 mL/min flow rate. N1 and N2 were purified using the same column equilibrated with 25 mM Tris-HCl pH 8.0, 150 mM NaCl, 2 mM $CaCl_2$ at 0.75 mL/min flow rate. NDV HN was purified using HiLoad Superdex 200pg 16-600 column (GE Life Sciences) equilibrated with 25 mM Tris-HCl pH 8.0, 150 mM NaCl at 1.0 mL/min flow rate. Gel filtration standard (Bio-Rad 1511901) was run using the same parameters. The final yields per L of Expi293 medium (Thermo Fisher) were 63 mg for H3 Vic, 32 mg for H3 Aichi, 2.3 mg for N1, 28 mg for N2, and 44 mg for NDV HN.

Expression and purification of SARS-CoV-2 RBD. SARS-CoV-2 RBD was expressed in Expi293F cells using ExpiFectamine293 transfection reagent and transfection enhancers (Thermo Fisher) as described above. SpyTag-RBD was purified from cell culture supernatant using Spy&Go affinity purification with minor modifications[53]. Briefly, filtered cell-culture supernatants were diluted with one third supernatant volume of TP buffer (25 mM orthophosphoric acid adjusted to pH 7.0 at 22 °C with Tris base) and adjusted to pH 7.2. Spy&Go resin equilibrated with the same buffer was mixed with the diluted supernatant and incubated at 4 °C for 1 h with gentle stirring. The mixture was poured into an Econo-Pak column (Bio-Rad) and the column allowed to empty by gravity. The resin was washed with 2 × 10 CV of TP buffer. SpyTag-RBD was eluted with 2.5 M imidazole in TP buffer adjusted to pH 7.0 at 22 °C. Elution buffer was added 1 CV at a time and incubated with the resin for 5 min before collecting each fraction. Elution fractions were analyzed using SDS-PAGE with Coomassie staining and the fractions containing SpyTag-RBD were pooled and dialyzed against 10 mM Tris-HCl pH 8.0, 200 mM NaCl. The sample was concentrated using Vivaspin-20 10 kDa and further purified using ÄKTA Pure 25 (GE Life Sciences) equipped with Superdex 75pg 16-600 column (GE Life Sciences), run at 1 mL/min. The dialysis buffer was used as the moving phase. The final yield of purified SpyTag-RBD per L of Expi293 medium was 99.6 mg.

Expression, purification and conjugation of antigens for human sero-reactivity ELISA. His_6-SpyCatcher, His_6-SpyCatcher003, His_6-SnoopCatcher, His_6-SUMO-SnoopTagJr, His_6-Spy0469 and His_6-PsaA were expressed in *E. coli* BL21(DE3) RIPL cells as described above for SpyCatcher003-mi3 with minor modifications. Protein expression was induced at OD_{600} = 0.5 with 420 µM IPTG and incubation was continued at 30 °C for 4 h. Cell pellets from 500 mL cultures were lysed in 50 mM Tris-HCl pH 7.8, 300 mM NaCl supplemented with 10 mM imidazole, the lysate was cleared by centrifugation, and the cleared lysate was loaded into gravity-driven polypropylene columns with Ni-NTA-sepharose (Qiagen) pre-equilibrated with the lysis buffer. Resin was washed 5 times with 10 CV of 50 mM Tris-HCl pH 7.8, 300 mM NaCl containing 10 mM imidazole and twice with 1 CV of 50 mM Tris-HCl pH 7.8, 300 mM NaCl with 30 mM imidazole. Bound proteins were eluted by 200 mM imidazole in 50 mM Tris-HCl pH 7.8, 300 mM NaCl and samples of each fraction were run on SDS-PAGE. Selected fractions were pooled and imidazole was removed by dialysis twice against 50 mM Tris-HCl pH 7.8, 300 mM NaCl. SnoopTagJr was released from His_6-SUMO-SnoopTagJr by incubation with lab-made His_6-tagged SUMO protease at 1:50 molar ratio for 13 h at 12 °C in the presence of 10% (v/v) glycerol. His-tags were removed from all other proteins by 16 h incubation at 4 °C with lab-made His_6-MBP-TEV protease added at 1:25 molar ratio in 50 mM Tris-HCl, 0.5 mM EDTA, 1 mM dithiothreitol (DTT) pH 8.0. His_6-MBP-TEV, His_6-tagged SUMO protease and the cleaved His_6 fragments were removed by recapturing with Ni-NTA-sepharose resin. Proteins in the flow-through were dialyzed against 50 mM Tris-HCl pH 8.5, 150 mM NaCl and stored at -80 °C.

Before human sero-reactivity ELISA, 50 µM SpyCatcher (cleaved with His_6-MBP-TEV protease) was reacted in PBS with two-fold molar excess of solid-phase synthesized SpyTag peptide (GAHIVMVDAYKPTK, 98% purity, Insight Biotechnology). 50 µM SpyCatcher003 cleaved with TEV protease was reacted in PBS with two-fold molar excess of solid phase-synthesized SpyTag003 peptide (RGVPHIVMVDAYKRYK, 95% purity, Insight Biotechnology). After 2 h incubation at 22 °C, excess Tags were removed by dialyzing three times against PBS pH 7.5 using 7 kDa M_W cut-off SnakeSkin dialysis tubing (Thermo Fisher). TEV protease-cleaved SnoopCatcher was reacted in 50 mM sodium borate pH 10.0 with 2-fold molar excess of SnoopTagJr peptide (GKLGSIEFIKVNKGS) generated by SUMO protease-cleavage of His_6-SUMO-SnoopTagJr as described above. SnoopCatcher and SnoopTagJr were

incubated for 16 h at 22 °C and excess SnoopTagJr was removed by repeated washing in a Vivaspin 500 spin column with 5 kDa M_w cut-off membrane. Complete Tag:Catcher conjugation and successful removal of excess tags were confirmed using SDS-PAGE with Coomassie staining.

Human sero-reactivity ELISA. For indirect ELISA, Maxisorp F96 plates (Nunc) were coated for 16 h at 4 °C with 100 μL 2 μg/mL of antigens diluted in PBS. Wells were washed 6 times with PBS supplemented with 0.05% (v/v) Tween-20 (PBS-T) and blocked with 300 μL Blocker Casein in PBS (Thermo Fisher) for 1 h at 22 °C, after which the wash with PBS-T was repeated. Serum samples were serially diluted into the blocking buffer using 7-point, 4-fold series starting at 1:100. 50 μL of serum samples were added per well and incubated for 2 h at 22 °C. Wells were washed 6 times with PBS-T and 50 μL alkaline phosphatase conjugated anti-human IgG antibody (Sigma-Aldrich A1543) diluted 1:1,000 in the blocking buffer was added to each well. After 1 h incubation at 22 °C, wells were washed 6 times with PBS-T with 3 min incubations at each wash. After the final wash, excess buffer was carefully removed by tapping the plates on paper towels. 100 μL 1 mg/mL para-nitrophenyl phosphate (pNPP) substrate (Sigma-Aldrich) diluted in 1 M diethanolamine (pH 9.8) was added to each well. OD_{405} was measured at 30 s intervals for 30 min using a Tecan Infinite F50 plate reader. To minimize plate-to-plate variation, control wells on all plates were coated with Spy0469 and incubated with a pool of 10 serum samples diluted 1:100. OD_{405} of these control wells was used to determine plate-specific time-points for data analysis. To correct for antigen-specific background caused by any non-specific binding of the secondary antibody, the mean OD_{405} of antigen-coated blank wells was subtracted from OD_{405} of wells incubated with serum samples. To correct for sample-specific background, each serum dilution was incubated on non-coated control wells and the blank-corrected OD_{405} from these wells was subtracted from OD_{405} of all antigen-coated wells incubated with the matching serum dilution. Endpoint dilution was defined as the intersection of the dilution curve and the horizontal line at OD_{450} = mean + 5× s.d. of antigen-coated blank wells. For correlation analysis of endpoint values, Pearson correlation coefficient was calculated in GraphPad Prism 7 (GraphPad software) using $\log_{10}$-transformed endpoint titers. Linear regression lines were fitted to the endpoint titers in GraphPad Prism 7 (GraphPad software) for data visualization.

For sandwich ELISA, Maxisorp F96 plates (Nunc) were coated for 16 h at 4 °C with 100 μL anti-His_6 antibody (Qiagen 34660) diluted to 0.3 μg/mL in PBS. Wells were washed 6 times with PBS-T, blocked with Blocker Casein in PBS (Thermo Fisher) and 100 μL of SpyTag:SpyCatcher, SpyTag:His_6-SpyCatcher, SpyTag003:SpyCatcher003 or SpyTag003:His_6-SpyCatcher003 at 2 μg/mL in the blocking buffer were added to each well. Plates were incubated for 1 h at 22 °C and washed with PBS-T as above. 50 μL of human serum samples diluted 1:100 in the blocking buffer were added to each well and plates were incubated for 2 h at 22 °C. The presence of SpyTag:SpyCatcher-specific antibodies was detected using alkaline phosphatase conjugated anti-human IgG antibody (Sigma-Aldrich, A1543) and pNPP substrate (Sigma-Aldrich) as described above for endpoint ELISA. To correct for sample-to-sample variance in non-specific binding, OD_{405} of control wells coated with anti-His_6 antibody and incubated with tagless SpyTag:SpyCatcher or tagless SpyTag003:SpyCatcher003 was subtracted from OD_{405} of the wells coated with anti-His_6 antibody and incubated with SpyTag:His_6-SpyCatcher or SpyTag003:His_6-SpyCatcher003, respectively.

The study was conducted according to the principles of the Declaration of Helsinki and in accordance with Good Clinical Practice (GCP). The analyzed samples were collected from healthy UK adults as a part of the VAC063 study (Clinicaltrials.gov identifier NCT02927145), University of Oxford, and used where informed, signed consent by the donor covered further use of the samples in vaccine research. The VAC063 study received ethical approval from the

UK NHS Research Ethics Service (Oxfordshire Research Ethics Committee A, Ref 16/SC/0345).

VLP thermal stability assay. To analyze VLP thermal stability, 50 μL aliquots of SpyCatcher-mi3 or SpyCatcher003-mi3 at 30 μM in 25 mM Tris-HCl, 150 mM NaCl, pH 8.5 were incubated at 25 °C, 37 °C, 55 °C, 75 °C or 95 °C for 60 min and cooled to 4 °C for 10 min using a Thermal Cycler C1000 (Bio-Rad). Following incubation, aggregates were removed by centrifugation at 16,900 g for 30 min at 4 °C. Samples of supernatant were resolved on 16% SDS-PAGE stained with InstantBlue Coomassie (Expedion) and imaged using a ChemiDoc XRS imager. The relative amount of soluble protein was analyzed using gel densitometry in ImageLab (version 5.2) software (Bio-Rad). The mean band intensities for samples incubated at 37 °C, 55 °C, 75 °C or 95 °C were normalized by dividing by the mean band intensity of samples incubated at 25 °C. All samples were analyzed in triplicate and plotted as mean ± 1 standard deviation.

VLP:antigen conjugation and solubility assay. Antigens at 2 μM, 3 μM, 4 μM or 6 μM were conjugated with 2 μM SpyCatcher003-mi3. SARS-CoV-2 RBD, OspC, NDV HN, H3 Vic and N2 were conjugated in 25 mM Tris-HCl, 150 mM NaCl, pH 8.0. H3 Aichi was conjugated in 25 mM Tris-HCl, 620 mM NaCl, pH 8.0. N1 was conjugated in 25 mM Tris-HCl, 150 mM NaCl, 2 mM EDTA, pH 8.0. β-galactosidase was conjugated in 25 mM Tris-HCl, 620 mM NaCl, 10 mM TCEP, pH 8.0. All conjugations were incubated at 4 °C for 16 h and possible aggregates removed by centrifugation at 16,900 g for 30 min at 4 °C. Samples of the supernatant were mixed with reducing 6× loading dye [0.23 M Tris-HCl, pH 6.8, 24% (v/v) glycerol, 120 μM bromophenol blue, 0.23 M SDS, 0.2 M dithiothreitol] and resolved on 12%, 14% or 16% SDS-PAGE using the XCell SureLock system (Thermo Fisher). Gels were stained with InstantBlue Coomassie (Expedion) and imaged using a ChemiDoc XRS imager (Bio-Rad). The intensities of bands on each lane were quantified using ImageLab (version 5.2) software (Bio-Rad) and Fiji distribution of ImageJ (version 1.51n). For each antigen, the mean intensities of all bands were normalized by dividing by the mean intensity of non-conjugated SpyCatcher003-mi3 resolved at 2 μM on the same gel. The normalized intensities of all bands on a single lane were plotted as stacked columns. Samples were analyzed in triplicate and plotted as mean ± 1 standard deviation.

For the solubility analysis of antigen-decorated VLPs, antigens were conjugated with SpyCatcher003-mi3 VLP at 1:1 molar ratio as described above. After conjugation, total protein samples were mixed with reducing 6× loading dye and resolved on 12-16% SDS-PAGE without removing possible VLP aggregates. Soluble protein samples were centrifuged at 16,900 g for 30 min at 4 °C to pellet any aggregates and the supernatants were resolved on 12-16% SDS-PAGE in the same way as total protein samples. Gel densitometry was used to determine the band intensity of non-coupled SpyCatcher003-mi3 on each lane as described above. Soluble protein fraction for each antigen-VLP conjugate was defined as the ratio of mean SpyCatcher003-mi3 band intensity for the soluble protein sample divided by the mean band intensity for the total protein sample. All samples were analyzed in triplicate and plotted as mean ± 1 standard deviation.

Dynamic Light Scattering (DLS). SpyTag-antigens were conjugated at 1:1 molar ratio with 2 μM SpyCatcher003-mi3 as described above. Samples were centrifuged for 30 min at 16,900 g at 4 °C to pellet possible aggregates and 30 μL was loaded into a quartz cuvette. Before each measurement, the cuvette was incubated in the instrument for 5 min to allow sample temperature to stabilize. All samples were measured at 20 °C using an Omnisizer (Viscotek) with 20 scans of 10 s each using 50% laser intensity, 15% maximum baseline drift and 20%

spike tolerance. The intensity of the size distribution was normalized to the peak value and plotted in GraphPad Prism 7 (GraphPad Software).

Thermofluor assay. SpyTag003-OspC or NDV HN-SpyTag were conjugated with SpyCatcher003-mi3 for 4 h at 22 °C, using both antigens and VLP each at 15 µM monomer concentration. Remaining non-reacted SpyCatcher003 on the particles was blocked by adding solid-phase synthesized SpyTag003 peptide (Insight Biotechnology) at 22.5 µM for 2 h at 22 °C. Non-conjugated antigens and excess peptides were removed by dialysis against PBS pH 7.8 for 16 h at 4 °C using dialysis membrane with 1 MDa M_W cut-off (Spectrum Chemical). Control samples with free antigens were not incubated with free SpyTag003 but were dialyzed against the same buffer using dialysis membrane with 3.5 kDa M_W cut-off (Spectrum Chemical). Possible aggregates were removed by centrifugation for 30 min at 16,900 g at 4 °C and protein concentration was determined using BCA assay (Pierce). A negative control containing non-decorated SpyCatcher003-mi3 was incubated with SpyTag003 and dialyzed as described above. All samples were diluted with PBS pH 7.8 to 2.5 µM protein concentration and mixed with 5× SYPRO Orange (Thermo Fisher) in 50 µL. Samples were heated in an Mx3005P thermal cycler (Agilent) using 30 s at 25 °C, temperature ramping at 0.5 °C increments every 30 s for 150 rounds, and 30 s at 25 °C. SYPRO Orange fluorescence intensity was measured after each round using 492 nm excitation and 610 nm emission filters. The raw intensity values of SpyCatcher003-mi3 were subtracted from the values of antigen-decorated particles. Values of buffer controls with 5× SYPRO Orange were subtracted from the values of free antigens. Sample T_m value was defined as the peak value of the first derivative of background-corrected fluorescence intensity. All samples were analyzed in triplicate.

Transmission electron microscopy (TEM). SpyTag-antigens were conjugated at 1:1 molar ratio with 2 µM SpyCatcher003-mi3 as described above. Samples were centrifuged for 30 min at 16,900 g at 4 °C and diluted 1:5 using 25 mM Tris-HCl, 150 mM NaCl, pH 8.0. Protein samples were applied to glow-discharged carbon 200 mesh copper grids for 2 min and blotted with filter paper. Samples were stained with 2% (w/v) uranyl acetate for 10 s, blotted and air-dried. Grids were imaged in a FEI Tecnai T12 transmission electron microscope at 120 kV using a Gatan US1000 CCD camera.

Immunogen preparation for vaccination. 3 µM H3 Vic-SpyTag003 was conjugated with 2 µM SpyCatcher003-mi3 in 25 mM Tris-HCl, pH 8.0, 150 mM NaCl. 3 µM H3 Aichi-SpyTag003 was conjugated with 2 µM SpyCatcher003-mi3 in 25 mM Tris-HCl, pH 8.0, 620 mM NaCl. 4 µM SpyTag-N1 was conjugated with 2 µM SpyCatcher003-mi3 in 25 mM Tris-HCl, pH 8.0, 150 mM NaCl, 2 mM EDTA. Reactions were incubated for 16 h at 4 °C and centrifuged for 30 min at 16,900 g at 4 °C. Matching non-conjugated control antigens were diluted in the same buffers and incubated and centrifuged in the same way. The concentration of each sample was determined using BCA assay (Pierce) and the immunogens diluted to 4 µg/mL antigen concentration. Recombinant H3 Vic and H3 Aichi immunogens were diluted using 25 mM Tris-HCl, 150 mM NaCl, pH 8.0. Recombinant N1 immunogens were diluted using 25 mM Tris-HCl, 150 mM NaCl, 1 mM $CaCl_2$ pH 8.0. The endotoxin content of all recombinant antigens was measured using the Limulus amebocyte lysate (LAL)-based Chromogenic Endotoxin Quantitation Kit (Thermo Fisher) following manufacturer's instructions. For all immunogens, measured endotoxin content was < 0.2 EU/mL. Immunogens were prepared within 2 days of mouse immunization and stored on ice. Before intramuscular (i.m.) immunization, immunogens were mixed 1:1 (25 µL + 25 µL) with AddaVax adjuvant (Invivogen).

S-FLU and Influenza virus production. Pseudotyped S-FLU viruses were generated with H1N1 A/Puerto Rico/8/34 virus (Cambridge strain) (PR8) backbone as previously described[54]. Briefly, the HA signal sequence in the HA viral RNA was deleted, and the HA gene replaced by a transgene encoding enhanced green fluorescent protein (eGFP) inserted in the same reading frame. The S-FLU pseudotyped with different surface HA was propagated in MDCK-SIAT1 cells (Madin-Darby Canine Kidney cells stably transfected with human 2,6-sialyltransferase, SIAT1) stably expressing the HA of choice using a lentiviral transduction system (MDCK-HA). Cells were maintained in D10 medium [Dulbecco′s Modified Eagle Medium (DMEM) supplemented with 10% (v/v) fetal calf serum (Sigma-Aldrich, F9665), 2 mM glutamine, 100 U/mL penicillin and 100 µg/mL streptomycin]. In brief, S-FLU seed virus was produced via transient transfection in HEK293T using Lipofectamine 2000 (Thermo Fisher) as described[54] in the presence of 0.5 to 1 µg/mL tosyl phenylalanyl chloromethyl ketone (TPCK)-treated trypsin (Sigma-Aldrich, 4370285) in viral growth media (VGM; DMEM, 1% (w/v) bovine serum albumin (Sigma-Aldrich A0336), 10 mM HEPES buffer, penicillin (100 U/mL) and streptomycin (100 µg/mL)][54]. Supernatant containing seed virus was then propagated in MDCK-HA in the presence of TPCK-Trypsin (0.5 to 1 µg/mL) for 48 h.

Seed virus of NYMC X217 (H3N2 A/Victoria/361/2011) Influenza virus was obtained from the National Institute for Biological Standards and Control (NIBSC, UK). X217 was propagated by infecting a monolayer of MDCK-SIAT1 with 10^3 - 10^4 infective units of X217 seed virus for 1 h before replacing with VGM containing 0.5-1 µg/mL TPCK-treated trypsin. Virus was harvested after 48 h.

S-FLU were quantified as 50% Culture Infectivity Dose (CID_{50}) with slight modifications to the published protocol[55]. X217 was quantified as hemagglutination unit (HAU). Briefly, harvested supernatants containing S-FLU were titrated in 2-fold serial dilution in VGM across a flat-bottom 96-well plate seeded with 3×10^4 MDCK-SIAT1 cells. The plate was incubated at 37 °C for 16 h and fixed with 10% neutral buffered formalin (Sigma-Aldrich). eGFP signal was then read using a CLARIOstar plate reader (BMG Labtech). The dilution of virus giving 50% fluorescence signal (EC_{50}) was calculated by linear interpolation. CID_{50}/mL was calculated as EC_{50} dilution × the number of cells per well (3×10^4). For HAU, X217 was diluted on two-fold dilution series in 50 µL PBS in V-bottomed 96-well plates in duplicates. 50 µL of 0.5% (v/v) human red blood cells (RBCs) in PBS were then added to each well and the plate was incubated at 22 °C for 30 min to 1 h. RBCs were obtained from donors within the MRC Weatherall Institute of Molecular Medicine and used according to the Best Practice Guidance set by University of Oxford Research Ethics Committee. HAU was reported as the reciprocal of the dilution of virus in the last well with complete hemagglutination.

Mouse immunization and blood sampling. Animal experiments were performed according to the UK Animals (Scientific Procedures) Act Project License (PBA43A2E4) and approved by the University of Oxford Local Ethical Review Body. C57BL/6 female mice (5 weeks old at the time of experiment) were obtained from Envigo. Mice were housed in accordance with the UK Home Office ethical and welfare guidelines and fed on standard chow and water *ad libitum*. Isoflurane (Abbott) anesthetized mice were immunized on day 0 and day 14 i.m. with 50 µL S-FLU viruses or 0.1 µg of recombinant antigens. S-FLU was used at 2×10^6 CID_{50} dose diluted in VGM. All recombinant antigens were matched to 0.1 µg antigen dose. Animals were routinely monitored at least twice weekly by observation and weighing. Sera samples were obtained on day 42 via cardiac puncture of humanely sacrificed mice. The collected whole blood in Microtainer SST tubes (BD) was allowed to clot at 22 °C for 1- 2 h before spinning down at 10,000 g for 10 min. The clarified sera were transferred to fresh tubes and stored at -20 °C.

ELISA binding of sera to H3 or N1 expressed on cells. MDCK-SIAT1 cells (1×10^4/well) were seeded in a 96-well U-bottomed plate two days before the assay. Next day wild-type Victoria/361/2011 virus (for H3 Vic expression), X31 H3N2 S-FLU (for H3 Aichi expression) or H7N1 S-FLU virus (for N1 expression) at multiplicity of infection ~1 was added to the cells and incubated for 16 h. Serum samples were serially diluted in PBS + 0.1% (w/v) bovine serum albumin (dilution buffer) using 10-point, 2.5-fold series starting at 1:40. 50 µL of serum samples were added to cells washed with PBS and incubated for 1 h at 22 °C. Cells were washed with PBS and 50 µL horseradish-peroxidase (HRP) conjugated anti-mouse IgG antibody (Dako P0447) diluted 1:800 in the dilution buffer was added to each well. After 1 h incubation at 22 °C, wells were washed twice with PBS. The signal was then developed by adding BM Blue POD substrate (3,3′-5,5′-tetramethylbenzidine) (Roche 11484281001) for 5 min before stopping with 50 µL of 1 M H_2SO_4. Plates were read at OD_{450} using a CLARIOstar plate reader (BMG Labtech). Binding EC_{50} was derived by linear interpolation from neighboring points in the titration curve. IC_{50}/EC_{50} ratio was calculated by dividing the $\log_{10}$-transformed IC_{50} from the microneutralization assay or ELLA by the $\log_{10}$-transformed EC_{50} from the cell-based titering. Samples with below-threshold titers in either assay were omitted from the analysis.

Indirect ELISA for mouse sera. 96-well MaxiSorp microtiter plates (Thermo Fisher) were coated for 16 h at 4 °C with 100 µL recombinant Influenza antigens diluted to 5 µg/mL in PBS. Plates were washed 6 times with PBS-T and blocked with PBS-T with 5% (w/v) skim milk for 1 h at 22 °C. Plates were washed 6 times with PBS-T and incubated with serum samples diluted in 1% (w/v) skim milk in PBS-T. All samples were diluted on a 2.5-fold, 12-point series starting at 1:40 dilution and analyzed in duplicate. Plates were washed 6 times with PBS-T and incubated for 1 h at 22 °C with 100 µL of goat anti-mouse total IgG conjugated to alkaline phosphatase (Sigma-Aldrich, A3562) at 1:3,000 dilution in 1% (w/v) skim milk in PBS-T. The wells were washed 6 times in PBS-T with 3 min incubations at each wash. After the final wash, excess buffer was carefully removed by tapping the plates on paper towels. 100 µL of 1 mg/mL pNPP substrate (Sigma-Aldrich) diluted in 1 M diethanolamine (pH 9.8), was added to each well. The reactions were stopped after 6 min by the addition of 25 µL 3 M NaOH per well. Absorbance at 405 nm and 630 nm was measured using a FLUOstar Omega plate reader (BMG Labtech). 630 nm absorbance was subtracted from the 405 nm value to correct for well-specific background. To correct for antigen-specific background signal, the mean absorbance values of blank wells coated with recombinant antigens and incubated with secondary antibody were subtracted from the absorbance values of wells coated with the same antigen and incubated with serum samples. GraphPad Prism 7 (GraphPad Software) was used to obtain 4-parameter logistic fits to the background-corrected data and to calculate EC_{50}.

Microneutralization assays. Microneutralization assays were performed as described previously[54] with minor modifications. Mice serum samples were heat-inactivated at 56 °C for 30 min. Sera were two-fold serially diluted in PBS starting at 1:40 dilution in a flat bottomed 96-well plate and incubated with virus for 2 h at 37 °C with 5% (v/v) CO_2 in a tissue culture incubator. The virus dilution used for the microneutralization assays was pre-determined by performing two-fold serial dilution on MDCK-SIAT1 cells. The highest dilution of the virus giving maximum plateau infection was chosen for use in the microneutralization assay. Indicator MDCK-SIAT1 cells (3×10^4 per well) were added to the sera and virus mixture and incubated for 16 h. The suppression of infection was measured on formalin-fixed cells by detecting eGFP (for S-FLU) or nucleoprotein (NP) (for wild-type viruses) using a fluorescence plate reader (CLARIOstar, BMG Labtech) as described above. For NP staining, cells were

permeabilized with PBS containing 0.5% (v/v) Triton X-100 and 20 mM glycine and stained with 1 µg/mL dilution of in-house produced anti-NP antibody 2-8C[54] biotinylated with EZ-Link Sulfo-NHS-LC-Biotinylation Kit (Thermo Fisher, 21335). The detection was performed through secondary Alexa Fluor 647-conjugated streptavidin (Thermo Fisher, S21374).

Enzyme-linked Lectin Assay (ELLA). ELLA assays were performed as previously described[56]. In brief, the two-fold serially-diluted heat-inactivated sera were incubated with pre-titrated A/England/195/2009 virus as the NA source for 1-2 h. The virus dilution for the assay was pre-determined by titrating to obtain at least 5-fold signal to noise ratio. The mixture was transferred to a Nunc Immunoassay ELISA plate (Thermo Fisher, 439454) pre-coated for at least 2 h with 25 µg/mL fetuin (Sigma-Aldrich, F3385). The plate was incubated for 18-20 h at 37 °C with 5% (v/v) CO_2 in a tissue culture incubator. The inhibition of NA enzymatic activity was detected by adding HRP-conjugated peanut agglutinin (Sigma-Aldrich, L7759) at 1 µg/mL after washing the plates 3 times with PBS and then developing with 50 µL o-phenylenediamine dihydrochloride (OPD) solution (Sigma-Aldrich, P9187). The enzymatic reaction was stopped after 5-15 min using 50 µL 1 M H_2SO_4 and the absorbance was read at 492 nm using a CLARIOstar plate reader (BMG Labtech).

Statistical analysis. All statistical analyses were performed using GraphPad Prism 7 (GraphPad Software). Comparisons between two groups were made using two-tailed Student's t-test when data normal distribution was assumed and Mann-Whitney U test when normal distribution could not be assumed. For pairwise comparison of SpyTag:SpyCatcher and SpyTag003:SpyCatcher003 endpoint titers in Fig. 2, Wilcoxon matched-pairs signed rank test was used. For all immunological assays in Fig. 5, statistical analyses were performed on $\log_{10}$-transformed data using one-way ANOVA with Bonferroni correction. Pearson correlation coefficient (PCC) of $\log_{10}$-transformed data was used to analyze the correlations between the endpoint titers of SpyTag:SpyCatcher, SnoopTag:SnoopCatcher and Streptococcal control antigens in Fig. S2 and S3. In all statistical tests, $p < 0.05$ was considered to be statistically significant.

Structure visualization. Protein structures were visualized in PyMOL version 2.2.2 (Schrödinger). Structural models are from the following PDB IDs: SpyTag:SpyCatcher 4MLI, SnoopTag:SnoopCatcher (RrgA D4 domain) 2WW8, RBD 6W41, OspC 1G5Z, NDV HN 3T1E, HA H3 4UO0, NA N1 2HTY, VASP tetramerization domain 1USE, β-galactosidase 6DRV. The SpyCatcher003-mi3 model is based on the previously published i3-01 model[57] and SpyTag:SpyCatcher PDB ID 4MLI, with the connecting linker modelled in PyMOL. To present antigens at the same scale, all antigens were rendered in PyMOL using orthoscopic view. A red sphere was added at the final main-chain atom where SpyTag or SpyTag003 was fused.

Data availability

Amino acid sequences of SpyCatcher003-mi3 and all SpyTag-antigens are available in Table S1. The cDNA sequences of the used antigens are available in GenBank as described above under "Expression constructs". Enquiries for further information and request for resources and reagents should be directed to and will be fulfilled by the Lead Contacts, Mark Howarth or Alain Townsend: mark.howarth@bioch.ox.ac.uk, alain.townsend@imm.ox.ac.uk.

REFERENCES

[44] T. U. J. Bruun, A.-M. C. Andersson, S. J. Draper, M. Howarth, *ACS Nano* **2018**, *12*,

8855–8866.
[45] A. H. Keeble, P. Turkki, S. Stokes, I. N. A. Khairil Anuar, R. Rahikainen, V. P. Hytönen, M. Howarth, *Proc. Natl. Acad. Sci. U. S. A.* **2019**, *116*, 26523–26533.
[46] B. Zakeri, J. O. Fierer, E. Celik, E. C. Chittock, U. Schwarz-Linek, V. T. Moy, M. Howarth, *Proc. Natl. Acad. Sci.* **2012**, *109*, E690–E697.
[47] G. Veggiani, T. Nakamura, M. D. Brenner, R. V. Gayet, J. Yan, C. V. Robinson, M. Howarth, *Proc. Natl. Acad. Sci. U. S. A.* **2016**, *113*, 1202–1207.
[48] C. M. Buldun, J. X. Jean, M. R. Bedford, M. Howarth, *J. Am. Chem. Soc.* **2018**, *140*, 3008–3018.
[49] D. G. Edmondson, S. Prabhakaran, S. J. Norris, A. J. Ullmann, J. Piesman, M. Dolan, C. Probst, C. Radzimski, W. Sticker, L. Komorowski, *Clin. Vaccine Immunol.* **2017**, *24*, e00306-16.
[50] M. Fairhead, G. Veggiani, M. Lever, J. Yan, D. Mesner, C. V. Robinson, O. Dushek, P. A. van der Merwe, M. Howarth, *J. Am. Chem. Soc.* **2014**, *136*, 12355–12363.
[51] R. Assenberg, O. Delmas, S. C. Graham, A. Verma, N. Berrow, D. I. Stuart, R. J. Owens, H. Bourhy, J. M. Grimes, *Acta Crystallogr. Sect. F Struct. Biol. Cryst. Commun.* **2008**, *64*, 258–262.
[52] K. D. Brune, D. B. Leneghan, I. J. Brian, A. S. Ishizuka, M. F. Bachmann, S. J. Draper, S. Biswas, M. Howarth, *Sci. Rep.* **2016**, *6*, 19234.
[53] I. N. A. Khairil Anuar, A. Banerjee, A. H. Keeble, A. Carella, G. I. Nikov, M. Howarth, *Nat. Commun.* **2019**, *10*, 1–13.
[54] T. J. Powell, J. D. Silk, J. Sharps, E. Fodor, A. R. M. Townsend, *J. Virol.* **2012**, *86*, 13397–13406.
[55] J. Xiao, P. Rijal, L. Schimanski, A. K. Tharkeshwar, E. Wright, W. Annaert, A. Townsend, *J. Virol.* **2017**, *92*, e00941-17.
[56] P. Rijal, B. B. Wang, T. K. Tan, L. Schimanski, P. Janesch, T. Dong, J. W. McCauley, R. S. Daniels, A. R. Townsend, K.-Y. A. Huang, *J. Virol.* **2020**, *94*, e01182-19.
[57] Y. Hsia, J. B. Bale, S. Gonen, D. Shi, W. Sheffler, K. K. Fong, U. Nattermann, C. Xu, P.-S. Huang, R. Ravichandran, S. Yi, T. N. Davis, T. Gonen, N. P. King, D. Baker, *Nature* **2016**, *535*, 136–139.
